# Shifting priorities: highly conserved behavioral and brain network adaptations to chronic stress across species

**DOI:** 10.1038/s41398-017-0083-5

**Published:** 2018-01-22

**Authors:** Yuliya S. Nikolova, Keith A. Misquitta, Brad R. Rocco, Thomas D. Prevot, Annchen R. Knodt, Jacob Ellegood, Aristotle N. Voineskos, Jason P. Lerch, Ahmad R. Hariri, Etienne Sibille, Mounira Banasr

**Affiliations:** 1 Campbell Family Mental Health Research Institute of CAMH, Toronto, Canada; 2 Department of Pharmacology and Toxicology, University of Toronto, Toronto, Canada; 3 Laboratory of NeuroGenetics, Department of Psychology & Neuroscience, Duke University, Durham, NC USA; 4 Mouse Imaging Centre (MICe), Hospital for Sick Children, Toronto, Canada; 5 Department of Medical Biophyssics, University of Toronto, Toronto, Canada; 6 Department of Psychiatry, University of Toronto, Toronto, Canada

## Abstract

Parallel clinical and preclinical research have begun to illuminate the biological basis of stress-related disorders, including major depression, but translational bridges informing discrete mechanistic targets for intervention are missing. To address this critical need, we used structural MRI in a mouse model and in a large human sample to examine stress effects on brain structure that may be conserved across species. Specifically, we focused on a previously unexplored approach, whole-brain structural covariance, as it reflects synchronized changes in neuroanatomy, potentially due to mutual trophic influences or shared plasticity across regions. Using the unpredictable chronic mild stress (UCMS) paradigm in mouse we first demonstrate that UCMS-induced elevated behavioral emotionality correlates with increased size of the amygdala and other corticolimbic regions. We further identify focal increases in the amygdala’s ‘hubness’ (degree and strength) set against the background of a global stress-related loss of network clustering and modularity. These macroscopic changes are supported on the molecular level by increased postsynaptic density-95 protein in the amygdala, consistent with stress-induced plastic changes and synaptic strengthening. Finally, we provide clinical evidence that strikingly similar structural network reorganization patterns exist in young adults reporting high childhood trauma and increased mood symptoms. Collectively, we provide initial translational evidence for a conserved stress-related increase in amygdala-centered structural synchrony, as measured by enhanced structural covariance, which is paralleled by a decrease in global structural synchrony. This putative trade-off reflected in increased amygdala-centered plastic changes at the expense of global structural dedifferentiation may represent a mechanistic pathway for depression and related psychopathology.

## Introduction

Major depressive disorder (MDD) is a chronic debilitating illness characterized by low mood, anhedonia, and emotion dysregulation.^1^ Stress, particularly when chronic or experienced early in life, is a major risk factor for psychiatric illnesses including MDD^2^ and many of its comorbidities, such as anxiety.^3^ Despite these clear links, the molecular and circuit-level neural processes mediating this association have proven complex, multi-faceted, and difficult to identify. Rapid translation and synergy between research in preclinical models of stress-related psychopathology and clinical studies are essential for attaining a more detailed understanding of these disorders’ pathophysiology and for developing novel, biologically informed treatments.^4^

The corticolimbic circuit which supports stress responsiveness and environmental threat learning represents a highly conserved nexus ideal for translational research. Consistently dysregulated in MDD,^5^ this circuit includes the amygdala, the hippocampus, and regions of the prefrontal cortex (PFC). Multiple functional MRI (fMRI) studies report amygdala hyperactivity to threat-related signals,^6–9^ as well as alterations in amygdala functional connectivity patterns within and beyond the corticolimbic circuit in MDD.^10,11^ Importantly, threat-related amygdala hyperactivity is further associated with clinical and subclinical anxiety,^12,13^ which are frequently comorbid with and may increase risk for MDD.^14^ More recently, amygdala hyperactivity was shown to predict future risk for developing stress-related symptoms of depression and anxiety.^15^ Moreover, while structural MRI studies of the corticolimbic circuit report gray matter reductions in the hippocampus^16–19^ and various subdivisions of the PFC in MDD,^20,21^ amygdala volume studies yield mixed results,^22–24^ with a notable minority showing increased volumes in patients.^22,25^

Parallel studies in postmortem human brain tissue and rodent stress models have yielded complementary insight into the cellular mechanisms underlying some of these macroscopic alterations. Specifically, illness-related structural and functional changes in the human PFC and hippocampus were attributed to reductions in cell number or neuronal atrophy and dendritic shrinkage.^26–29^ Preclinical studies using chronic stress models describe similar dendritic reorganization and reduction in spine number in homologous brain regions.^30–36^ Conversely, in the amygdala, chronic stress exposure induces hypertrophy attributed to increased dendritic complexity and spine number.^34^

Research combining MRI-based assessment of brain structure with traditional molecular assays in rodent models, represents a particularly powerful translational platform to bridge these parallel lines of work. However, to date, only a few studies have used this strategy in the context of stress.^37,38^ Specifically, a recent study mapped social avoidance behavior onto volumetric changes in the amygdala, as well as other corticolimbic, basal ganglia and brainstem regions in a social defeat stress mouse model.^38^ Critically, this study also demonstrated stress-related alterations in the pairwise structural covariance among the hippocampus, cingulate cortex, and ventral tegmental area. Importantly, none of these prior studies have sought to integrate their results directly with a human sample.

Measures of structural covariance based on cross-region correlations in neuroanatomic properties are thought to reflect synchronized structural changes due to mutual trophic influences or shared experience-dependent plasticity among regions and networks.^39^ While such structural synchronization patterns map partially onto functional connectivity, they show distinct clustering and network properties and thus likely reflect complementary circuit properties.^40^ Although probing regional brain volume and pairwise structural covariance patterns in rodent models has provided partial insight into the functional impact of stress,^38^ investigating its effects on whole-brain structural covariance network patterns is apt to allow a more comprehensive characterization of novel mechanistic pathways of risk for stress-related depression and anxiety.

Here we conducted the first cross-species translational investigation of stress-related effects on MRI-assessed brain structure, focusing on whole-brain structural covariance patterns in a mouse model and a large human sample. First, using unpredictable chronic mild stress (UCMS), a widely adopted mouse model of stress-related pathophysiology with particularly strong face and construct validity,^41^ we investigated the proximal impact of UCMS on behavior, regional structural and synaptic changes, and brain network reorganization patterns. To establish their translational utility, we then sought to confirm the presence of similar structural covariance network changes in humans as a function of early-life stress, and as potential risk marker of depression.

## Methods

A complete description of all methods can be found in Supplementary Materials.

### Mouse sample

#### Subjects

Eight-week-old Balb/c mice (Charles River Laboratories, Quebec, Canada) were housed under normal conditions, with ad libitum access to food, water and a 12/12 light/dark cycle except when specified for the UCMS paradigm and for behavioral testing. Mice were exposed to UCMS for 5 weeks (3–4 randomized stressors/day) as in ref. 42 (*n* = 12/group). All animal procedures were performed in accordance with the Canadian Council on Animal Care (CCAC) guidelines.

### Human sample

#### Participants

A total of 1208 participants from the Duke Neurogenetics Study (DNS, 703 women, mean age 19.68 ± 1.26 years) had valid structural MRI and Childhood Trauma Questionnaire (CTQ^43^). The CTQ assesses the severity of five types of trauma: emotional abuse; physical abuse; emotional neglect; physical neglect; and sexual abuse. We selected for analysis participants scoring in the top 25% (>37, *n* = 299, 177 women) and bottom 25% (<27, *n* = 237, 143 women) of the observed range of total CTQ scores. All participants provided informed consent in accordance with Duke University guidelines.

### Mouse behavioral assessment

At the end of the UCMS protocol, mice were assessed on a series of behavioral tests for anxiety-like/depressive-like behavior (denoted as ‘behavioral emotionality’), including the elevated plus maze (EPM), open field (OF), forced swim test (FST), novelty suppressed feeding (NSF), sucrose consumption and cookie tests. We also assess the effects of UCMS in a novel paradigm allowing for repeated assessment of home-cage-like behavior and response to an anxiogenic stimulus.^42,44–46^

### MRI data acquisition and preprocessing

Mouse brains were perfused and scanned ex vivo on a multi-channel 7.0 Tesla MRI scanner (Varian Inc., Palo Alto, CA, USA) as previously described.^38^ For valid cross-species comparison, human MRI data processing was kept as similar as the processing applied to mouse imaging.

### Immunohistochemistry and microscopy analysis

Following MR imaging, 14 µm-thick mouse sections containing the basolateral amygdala were cryocut. Matched sections for anteroposterior level across animals and across groups were processed for immunohistochemistry to assess postsynaptic density protein-95 (PSD95), microtubule associated protein 2 (MAP2), and vesicular glutamate transporter (VGLUT1). VGLUT1 and PSD95 were chosen for this analysis because they represent pre- and post-synaptic markers, respectively, whose expression level is influenced by both synaptic activity and synapse number. MAP2 is a widely used marker of the dendritic neuronal compartment and was chosen to determine whether volume changes are linked to changes in dendritic complexity.

### Mouse behavior and cellular analysis

Weight, coat state, and performance in the phenotypers were measured weekly and data were analyzed using repeated measures ANOVA, followed by Bonferroni-corrected post hoc comparisons. Data in the classical behavioral tests were analyzed using *t*-tests. The behavioral data were summarized across multiple tests using a *z*-score methodology.^47^ Consistent behavioral dimensions were identified using a principal component analysis (PCA) on 34 major behavioral variables.

### Mouse and human volumetric MRI analysis

The volume of each ROI was compared between the UCMS-exposed and the control groups, using a general linear model with a false discovery rate (FDR) of *q* < 0.05. In the mouse, additional regression analyses examined the association between volume in each ROI and behavioral PC1.

### Cross-species structural covariance network analysis

Correlation matrices were created separately for the stress and control groups using 155 ROIs in the mouse and all 56 ROIs based on the Harvard-Oxford Atlas in the human sample. Consistent with prior work,^48^ negative correlations were discarded and correlation matrices were thresholded at a broad range of density thresholds (0.10–0.30), sequentially considering the top 10–30% strongest connections in increments of 1%. Each thresholded matrix was converted into a weighted graph. At each density threshold, we computed measures of network segregation (transitivity, clustering coefficient, and modularity) and integration (mean path length), separately for the control and stress groups. For each ROI showing an effect of stress, we computed degree centrality and strength metrics to assess changes in its position or ‘hubness’ in the network. For each threshold and metric, permutation testing (*n* = 10,000) was performed to obtain a null distribution, against which empirical two-tailed *p*-values were computed.

## Results

### Chronic stress alters home-cage-like behavior, increases response to anxiogenic challenge, and elevates overall behavioral emotionality

**Fig. 1:**
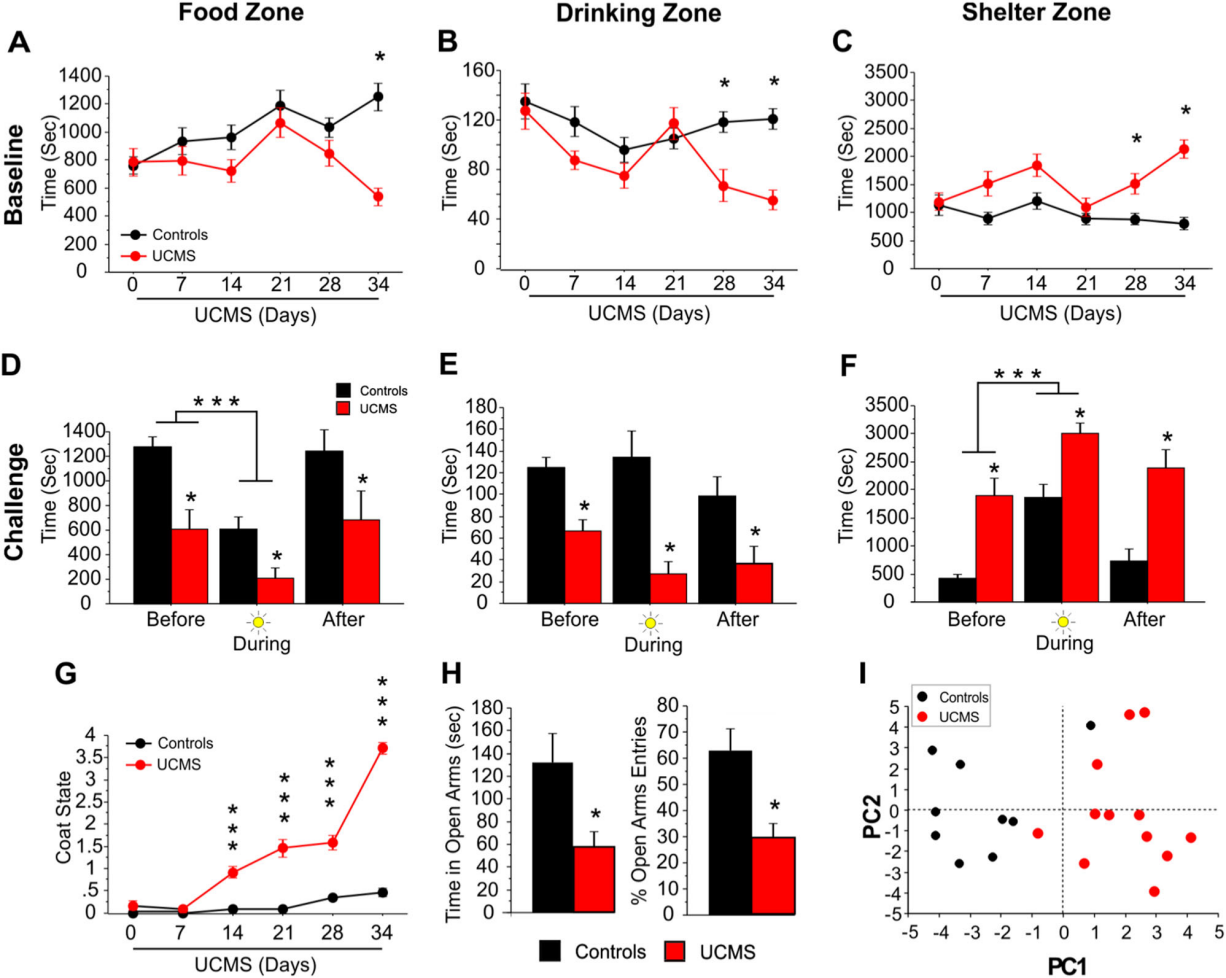
UCMS effects on behavior. **a**–**c** UCMS alters home-cage-like behavior. Data represent the average of the first 4 h of the dark cycle measured each week throughout the UCMS exposure (*n* = 12/group). UCMS-exposed mice showed a decrease in the time spent in the (**a**) food zone and the (**b**) drinking zone and an increase in time spent in the (**c**) shelter zone. Post hoc analysis showed that the effects all three measures were observed at the end of the UCMS exposure (*p < 0.05) compared to home cage controls. **d**–**f** Spotlight challenge induces an anxiogenic response in both control and UCMS-exposed mice. Data presented correspond to the time spent in each zone for the hour before, during and after the spotlight challenge on day 34. UCMS-exposed mice overall spent less time in the (**d**) food zone and (**e**) drinking zone, and more time in the (**f**) shelter zone. Both control and UCMS-exposed mice spent less time in the food zone during the light challenge (***p < 0.001) in favor of the shelter zone. Post hoc analysis showed that the UCMS mice are significantly different from controls, before, during and after the light challenge (*p < 0.05). **g** UCMS induces degradation of coat state quality. This effect was progressive starting at week 2 (post hoc ***p < 0.0001 compared to control group). **h** UCMS induces anxiety-like deficits in the elevated plus maze test. UCMS-exposed animals showed a significant decreased in time and percent entries in the open arms compared to the control group (*p < 0.05). Bar and linear graphs show mean ± s.e.m. **i** PC1 captures the variance attributed to UCMS. Principal component analysis revealed 3 components capturing behavioral variance across the UCMS and control animals. PC1 scores account for the highest between-group difference, relative to PC2 and PC3, thus likely capturing the effect of stress dimensionally across groups

Using a novel test monitoring home cage-like behavior (see Methods), we show that mice spent more time in the food and drinking zones early in the dark cycle, followed by greater time spent in the shelter later in the dark cycle (Supplementary Figure 1A–C). Four hours into the dark cycle, an anxiogenic spotlight challenge over the food was applied, prompting mice to avoid this zone and the nearby drinking zone in favor of the shelter zone (Supplementary Figure 1D–F). Before the challenge, UCMS-exposed mice showed a progressive preference for the shelter zone (*F*_5,110_ = 5.347; *p* < 0.001) and avoidance for the food (*F*_5,110_ = 6.193; *p* < 0.0001) and drinking (*F*_5,110_ = 4.356; *p* < 0.01) zones, compared to control mice (Fig. 1a–c). This difference became statistically significant starting in the fourth week (Fig. 1a–c; Post hoc *p* < 0.05), consistent with a chronic stress effect. This anxiogenic effect of UCMS is amplified during the spotlight challenge (*F*_1,22_ = 64.220, *p* < 0.0001, post hoc *p* < 0.05; Fig. 1d–f) over the course of stress exposure (Supplementary Figure 1).

Additionally, UCMS induced a progressive deterioration of coat state, significant starting the second week (Fig. 1g). UCMS effects were also measured on more classically used tests (Supplementary Figure 2). Specifically, UCMS-exposed mice displayed increased anxiety-like deficits in the EPM (Fig. 1h) and NSF tests (Supplementary Figure 2). Mixed results were obtained with other tests (Supplementary Figure 2). Collectively, however, after 5 weeks of UCMS, mice displayed higher behavioral emotionality scores across measures when compared to control mice (*p* < 0.0001, Supplementary Figure 2). A principal component analysis (PCA) of the behavioral data indicated that the first component (PC1) accounted for 22.59% of behavioral variance across groups, with highest loadings from stress-related variables from individual tests (Supplementary Figure 3). Accordingly, UCMS-exposed mice had significantly higher PC1 scores than controls (Fig. 1l; *p* < 0.0001), and similar scores for the next two PCs (Fig. 1i and Supplementary Figure 3). Hence, higher PC1 scores reflect stronger behavioral emotionality dimensionally across groups.

### Chronic stress in mice induces volumetric changes in corticolimbic circuitry

**Fig. 2:**
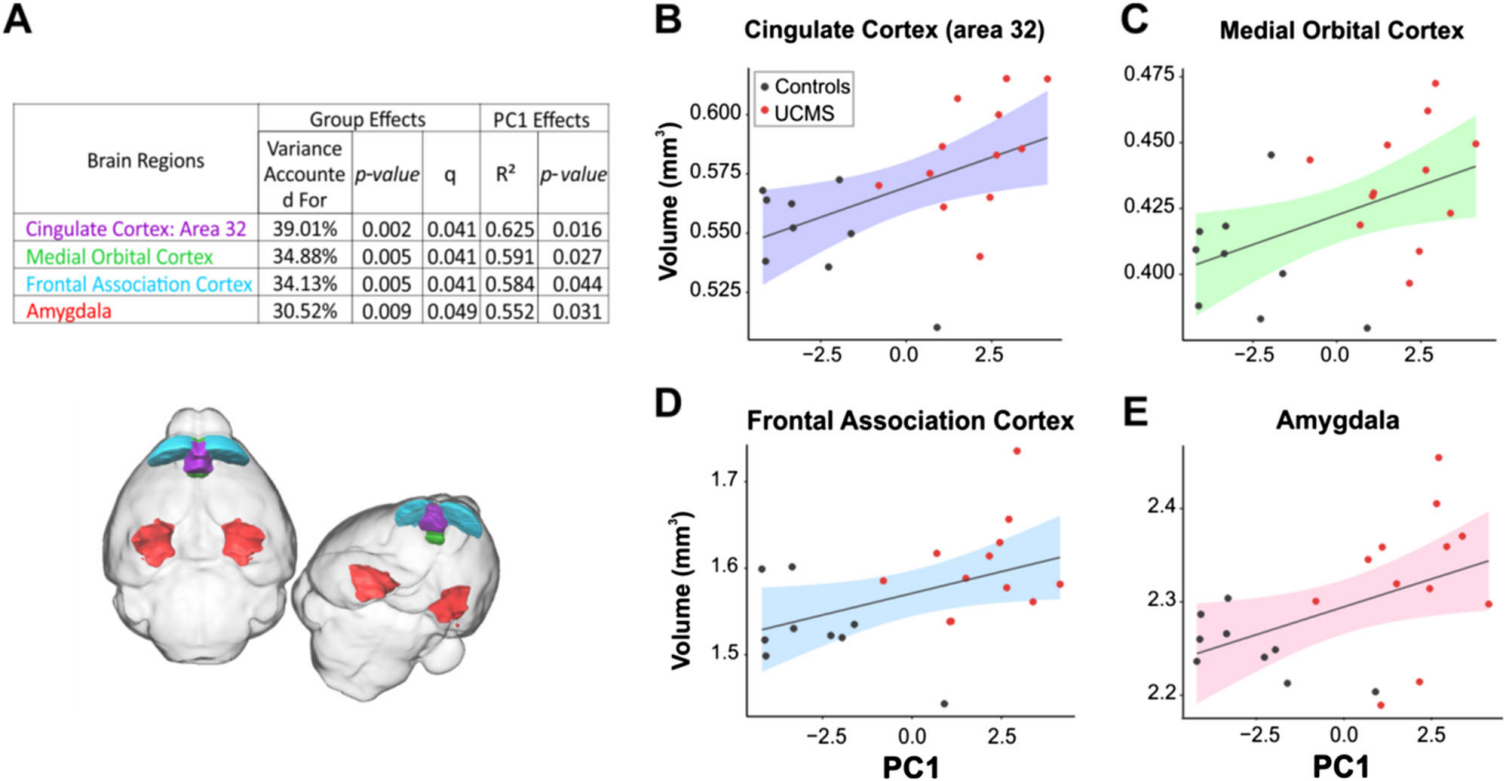
Four brain regions showed UCMS-induced changes in volumes that positively correlated with behavioral emotionality. **a** Table summarizes group and PC1 significant effects for each brain region significantly affected by UCMS, including variance accounted for, *p* values, *q* value following FDR correction for group effect on regional volume, and *r*^2^ and *p* values for correlation between volume and behavioral emotionality assessed by PC1. The four regions are similarly color-coded in the table and brain overlay figure. **b**–**e** Scatterplots depicting the significant positive correlation between behavioral emotionality (PC1) and brain volume for (**b**) cingulate area 32 (prelimbic cortex), (**c**) medial orbital cortex, (**d**) frontal association cortex, (**e**) amygdala. Colored bands represent 95% confidence intervals

We first focused the structural MRI analysis on UCMS effects on 26 a priori brain regions of broad cross-species relevance for emotion and behavioral regulation, comprising key nodes in the well-conserved corticolimbic, corticostriatal, and corticohippocampal circuits, as well as all association cortices. UCMS-exposed mice had larger volumes in corticolimbic circuit regions, including cingulate area 32 (prelimbic cortex) and amygdala as well as the frontal association, medial, and dorsolateral orbital cortices (Supplementary Table 1). In addition, we found positive correlations between UCMS-induced increases in volumes across regions, except the dorsolateral orbital cortex, and behavioral emotionality (indexed by PC1) (Fig. 2a–e). Therefore, these four regions were highlighted in subsequent structural covariance analyses. A whole-brain exploratory volumetric analysis of all 159 ROIs yielded no significant effects of UCMS surviving FDR correction for multiple comparisons (*q* > 0.05), although pre-selected MDD-relevant regions were overrepresented among the top 20 strongest associations (*χ*^2^ = 19.85, *p* = 0.0001; Supplementary Table 2). UCMS had no effect on total brain volume (*p* = 0.63).

### Chronic stress in mice induces structural covariance network adaptations featuring increased amygdala hubness

**Fig. 3:**
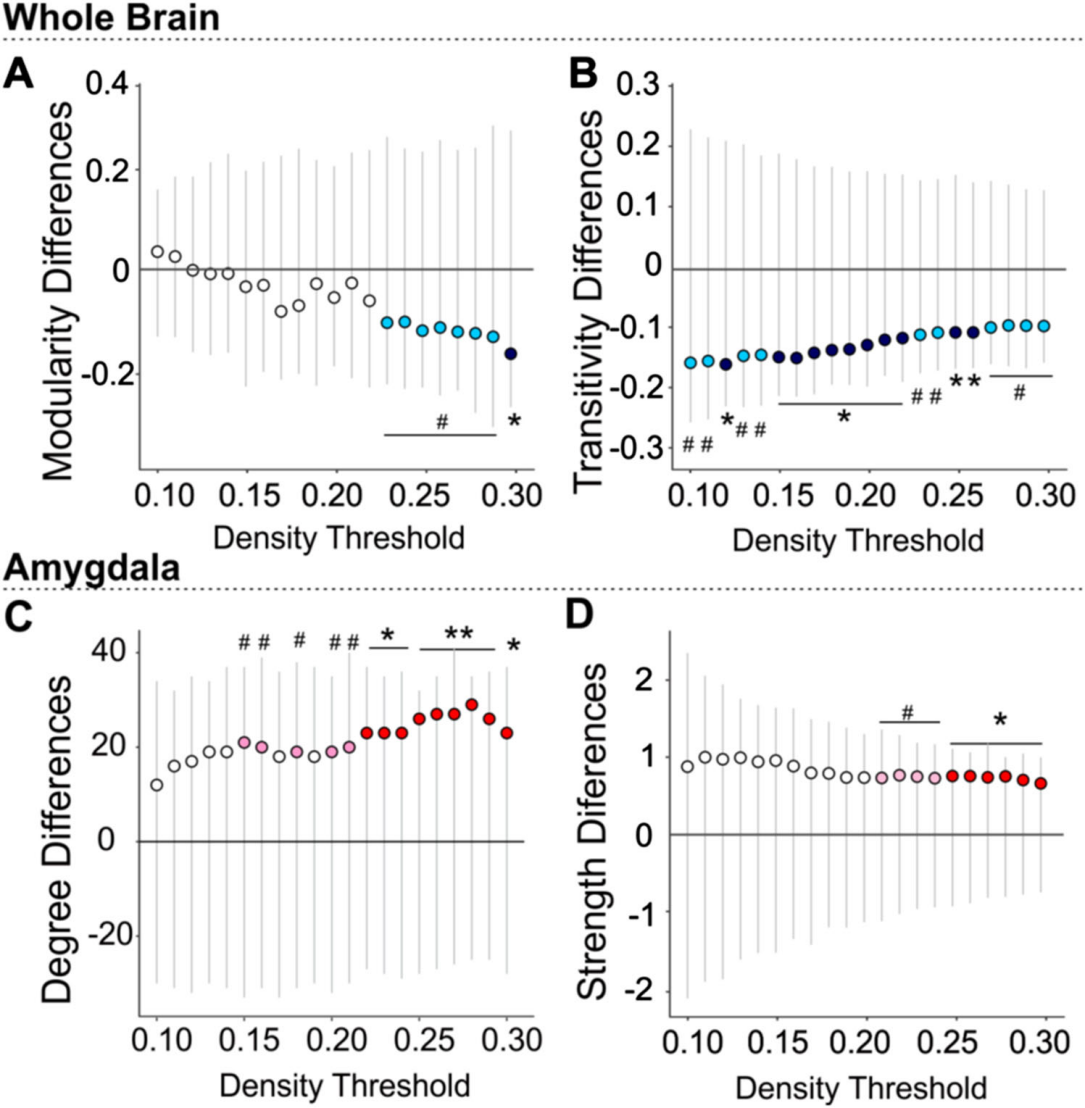
UCMS-induced changes in the whole structural covariance-based brain network are accompanied by amygdala changes in degree and strength within the network, as well as synaptic protein density. Permutation results showed significantly reduced whole-brain network **a** modularity and **b** global transitivity as well as an increase in (**c**) amygdala degree and **d** amygdala strength in UCMS mouse brains when compared to control. Gray lines represent the spread of the null distribution of between-group differences in each metric, obtained from 10,000 permutations at each density threshold tested. The circles represent the observed between-group differences (UCMS vs. control). Circles below the 0 line indicate the metric is lower in the UCMS group and are colored in dark or light blue, if the difference is significant (*P < 0.05, **P < 0.01) or trending (^#^*P* < 0.1), respectively. Circles above the horizontal *y* = 0 line, indicate the metric is higher in the UCMS group compared to control. Significant differences are marked as *P < 0.05, **P < 0.01 (red) and trends as # *P* < 0.1 (pink)

Using whole-brain graph theory structural covariance analysis on all 155 ROIs (ventricles excluded), we further investigated UCMS effects on measures of global network clustering and modularity (broadly indicative of capacity for specialized local processing), and mean path length (broadly indicative of integration capacity across the whole-brain). We then compared groups on node-level measures of ‘hubness’ or centrality (degree, strength, betweenness, and closeness centrality) computed for the four regions showing strongest links to stress and behavioral emotionality in our volumetric analyses. Consistent with prior work,^49,50^ we tested these effects across a series of network density thresholds, considering an increasing proportion of the networks’ strongest connections (10–30% range, in increments of 1%). UCMS induced marked disruptions of network clustering, as indexed by reductions of transitivity and modularity, when compared to the control group. UCMS network clustering was lower across most thresholds and reached peak significance in the 15–22% (transitivity, *p* < 0.05) and 29–30% density ranges (modularity, *p* < 0.05; Fig. 3a, b). Area under the curve (AUC) analyses confirmed aggregate significance across all thresholds (*p* < 0.001).

Against this background of weakened global clustering and modularity associated with UCMS, the amygdala showed a consistent increase in ‘hubness,’ reflected by higher degree centrality and strength, reaching peak significance in the 22–30% and 25–30% (Fig. 3c, d) density ranges, respectively, and accompanied by higher closeness centrality (i.e., distance to all other network nodes) at 22% density. More precisely, in the UCMS group, the amygdala gained structural covariance with multiple brain regions including areas involved in behavioral output, fear learning, and the fight-or-flight response (Supplementary Table 3) elevating its degree rank within the network from 60 to 2 (out of 155 regions) (Supplementary Table 4). Furthermore, the decrease in modularity and increase in amygdala degree were proportional to between-group behavioral emotionality differences (Supplementary Figure 4A, C). No other ROIs with UCMS-related volumetric changes showed significant differences in degree, strength or centrality according to our a priori criteria (see Methods). No differences in average path length emerged (all *p* > 0.2), suggesting UCMS-induced network changes may be confined to disruption of local processing, rather than global integration capacity.

### Chronic stress in mice induced an increase in amygdala PSD95 puncta density

**Fig. 4:**
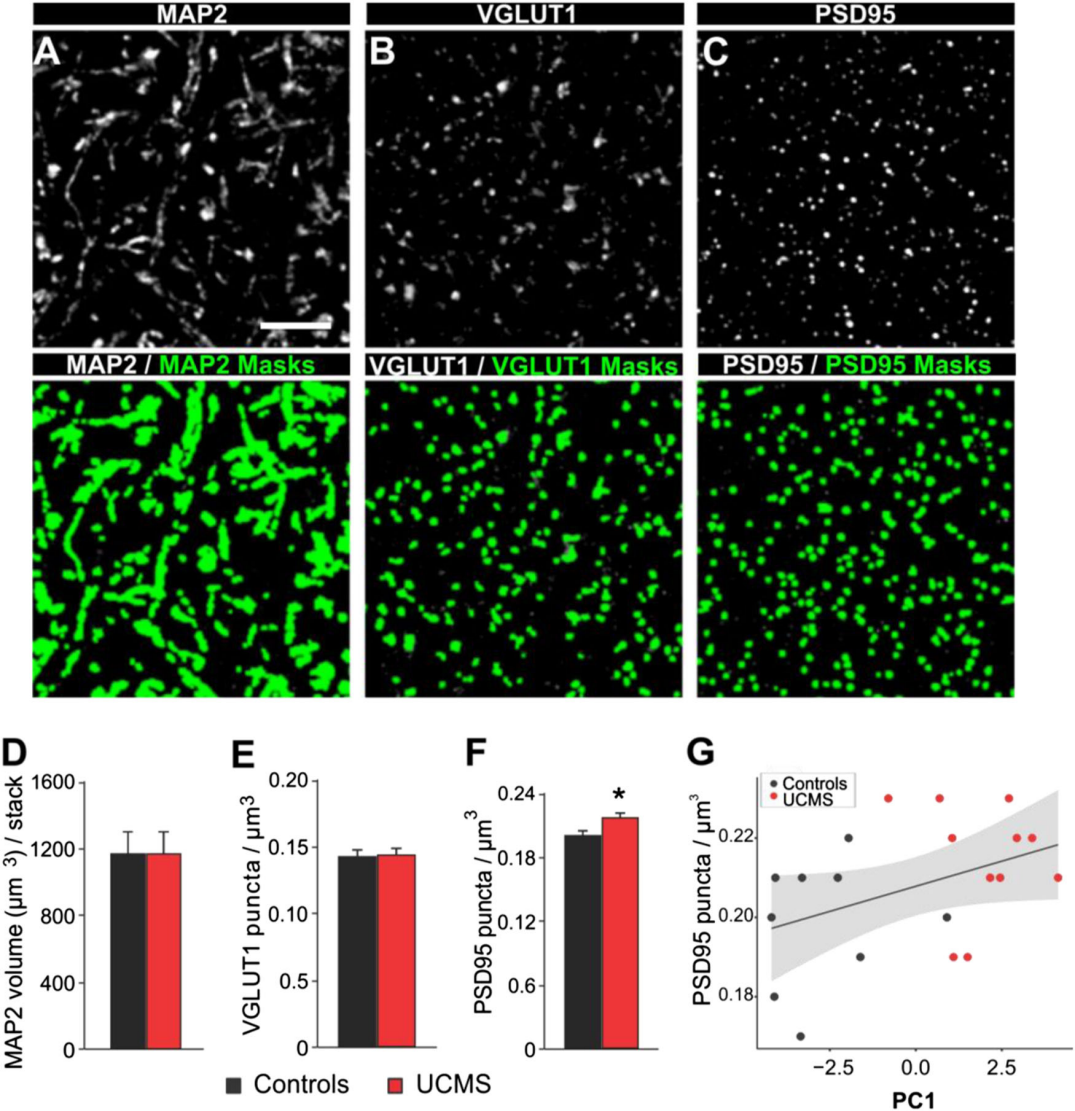
UCMS-induced changes in synaptic marker densities. Projection images (i.e., three-dimensional composites or z-stack of three *z*-planes separated by 0.25 µm) of a mouse basolateral amygdala immunolabeled for (**a**) MAP2, (**b**) VGLUT1 and (**c**) PSD95 showing single channels (above black and white) and single channels overlaid with corresponding object masks for each marker (below in green). Scale bar = 5 µm. While UCMS induced no significant changes in (**d**) MAP2 volume (µm^3^) per stack or (**e**) in VGLUT1 puncta per µm^3^, UCMS increased **f** the number of PSD95 puncta per µm^3^. Bar graphs show mean ± s.e.m. *P < 0.05 compared to control group. **g** PSD95 density in the amygdala showed a trending positive correlation with behavioral emotionality across groups indexed by PC1. Gray bands represent 95% confidence intervals

Given the role of the amygdala in UCMS-induced behavioral and imaging results, we next investigated the changes in expression of markers of pre-synaptic and post-synaptic compartment activity (VGLUT1 (vesicular glutamate transporter 1) and PSD95 (postsynaptic density protein 95), respectively) and dendritic structure (microtubule-associated protein 2 or MAP2^51^) in the basolateral amygdala of the same UCMS-exposed mice. Quantitative microscopy revealed higher density of PSD95 puncta in the UCMS group, relative to controls (Fig. 4a–g). Furthermore, PSD95 puncta density in the basolateral amygdala showed a trending positive correlation with behavioral emotionality across groups as indexed by PC1 (*R*^2^ = 0.174; *p* = 0.067; Fig. 4g). In contrast, there were no significant effects of UCMS on the densities of the dendritic marker MAP2 and presynaptic marker VGLUT1.

### Individuals exposed to early life stress exhibit structural covariance network adaptations and elevated mood symptoms mirroring UCMS-exposed mice

**Fig. 5:**
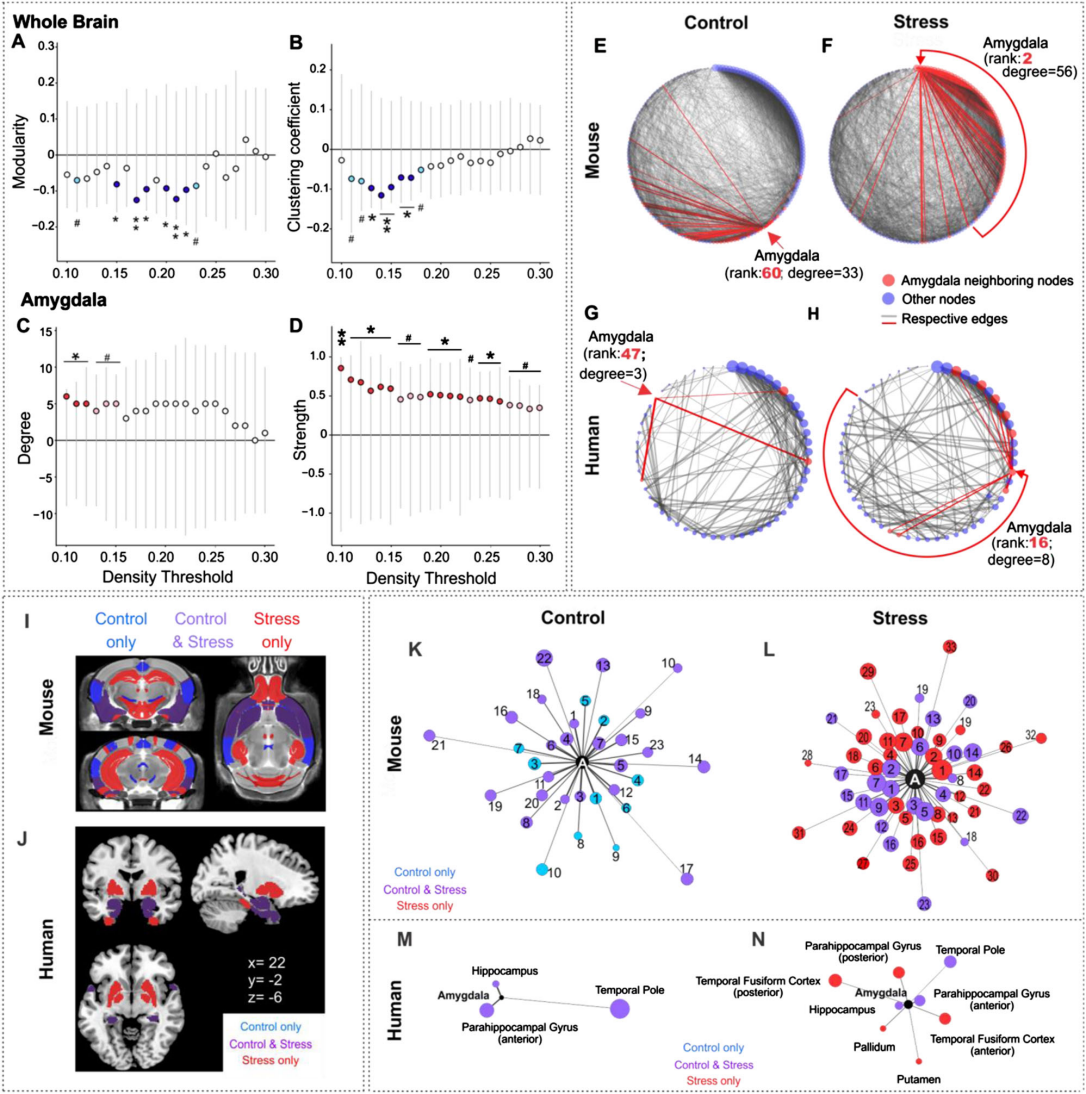
Cross-species convergence in brain network adaptations to chronic stress. Reductions of whole brain network modularity (**a**) global clustering coefficient (**b**) and increases in amygdala degree (**c**) and amygdala strength (**d**) were found in individuals with childhood trauma compared to a control group (low vs. high CTQ). Statistically different decreases are colored in dark (*p < 0.05) or light blue (^#^*p* < 0.1), and increases in red and pink, respectively. **e**–**h** Circular network plots depicting the mouse structural covariance network at 22% density (the lowest density where between-group differences emerge) in control (**e**) and stress (**f**) conditions. **g**, **h** Human networks depicted at 12% density in (**g**) control (or low CTQ) and (**h**) stress (or high CTQ) conditions. Nodes are arranged by degree clockwise, with highest-degree node on top of the circle. Degree is further reflected in node size. The amygdala and its direct connections are highlighted in red. **i**, **j** Anatomical view of the stress effects on the amygdala structural covariance subconnectome in both human and mouse brain. Overlays represent binarized masks onto select structural MR image slices of **i** mouse (coronal slices: Bregma −2.10 mm (top), −3.20 mm (bottom)) and (**j**) human brain (MNI coordinates: *x* = 22, *y* = −2, *z* = −6). **k**–**n** Edge-weighted spring-embedded layout network diagram of the amygdala′s direct structural covariance connections across species (mouse—k, l; human—m, n) and conditions (control—k, m; stress l, n). In panels **i**–**n**, regions in purple are part of the amygdala’s network common to control and stress groups and in blue or red are regions unique to each group. Node size reflects overall number of connections for that node within the whole network, however only the connections with the amygdala are pictured. A full list of all visualized nodes is provided in Supplementary Tables 3, 4 and 7)

We next turned to a human imaging study to investigate clinical evidence of similar network reorganization patterns. In this sample, individuals with greater experience of early life stress as indexed by the Childhood Trauma Questionnaire^43^ (top 25%, CTQ > 37; *n* = 299) reported higher levels of current depressive and anxiety symptoms, relative to individuals with lower early stress (bottom 25%, CTQ < 27, *n* = 237; *p* < 0.001). These individuals were also significantly more likely to have been diagnosed with a current or past major depressive disorder (Supplementary Table 5), and had lower total brain volume (*p* = 0.002, adjusted for sex; data not shown). Local volumetric comparisons revealed this group also had relatively larger occipital fusiform gyrus (*t*(534) = 4.26, *p* < 0.001, *q* = 0.001, FDR-corrected, normalized by whole-brain volume), but no other regional differences emerged after correcting for multiple testing (Supplementary Table 6). In contrast, structural covariance analysis revealed a pattern strikingly similar to the one observed in the UCMS model, where higher early stress was associated with reduced network clustering (measured by global clustering coefficient) and modularity (*p* < 0.05**;** Fig. 5a, b) as well as increased amygdala degree centrality and strength (*p* < 0.05; Fig. 5c, d, Supplementary Table 7), proportional to between-group differences in self-reported depressive symptoms (Supplementary Figure 4D). In further parallel to UCMS (Fig. 5e, f, k, l), in the higher early stress group, the amygdala increased its structural synchrony with brain areas implicated in behavioral output (pallidum and putamen) alongside more extensive subregions of the temporal lobes, elevating its degree rank within the network from 47 to 16 (out of 56 regions; Fig. 5g, h) and expanding its immediate neighboring nodes (Fig. 5j, m, n).

## Discussion

Using a novel translational approach centered on parallel applications of structural neuroimaging, we provide initial cross-species evidence for large-scale behavioral and brain network alterations associated with chronic stress. Specifically, mice exposed to chronic stress exhibited elevated anxiety-like behavior and overall behavioral emotionality across multiple tests. In addition, UCMS-exposed mice showed volumetric increases in corticolimbic circuit regions supporting stress responsiveness and threat learning, such as the prelimbic cortex and amygdala. We further discovered uniquely increased structural covariance between the amygdala and multiple regions, against a background of globally reduced network clustering and modularity. The increased amygdala structural covariance in UCMS-exposed mice was further represented at the cellular level as increased postsynaptic protein PSD95 immunoreactivity. Remarkably, we observed similar amygdala-centered alterations in structural covariance networks in individuals having been exposed to early life stress and experiencing greater symptoms of depression and anxiety in early adulthood.

Previous work using the UCMS model has reported elevated behavioral emotionality as assessed with classical paradigms measuring anxiety-like and anhedonia-like deficits.^42^ We confirmed that UCMS induced such anxiety-like deficits in the EPM and NSF tests, whereas other tests showed no changes. Furthermore, using a novel measure of home cage-like behavior we found that UCMS-induced behavioral changes manifested by a marked preference for the animal to hide in the shelter and avoid the food and drinking zones, both at baseline and during an anxiogenic spotlight challenge. We interpret these behavioral changes as stress-induced hiding and/or avoidance behavior similar to those elicited by classical behavioral tests such as the EPM, OF, and NSF. Additionally, this novel paradigm also allows for monitoring the animals’ behavior on a weekly basis, revealing that the preference for the shelter zone amplified over the course of UCMS and confirming the paradigm’s validity as a repeatable measure of chronic stress-related changes in anxiety-like behavior. Unlike the EPM, OF, and NSF tests, this test is automatized, involves little experimental or experimenter intervention, does not require the environment to be novel or anxiogenic, and therefore represents an important new tool for assessing longitudinal changes in behavior in stress-based models.

At the neural circuit level, we found increased volumes of the amygdala as well as the prelimbic, frontal association, and medial orbital cortices following UCMS that were correlated with behavioral emotionality. The increase in amygdala and prelimbic cortex volume is consistent with prior findings of amygdala hypertrophy in other stress-based rodent models^34,52^ as well as the critical role of the amygdala in stress responsiveness and mood pathophysiology.^53,54^ While amygdala volumetric findings in human neuroimaging are mixed,^23,24^ several studies suggest larger amygdala volumes may be present in early-course depression (as modeled here by 4–5 weeks of stress exposure in mice), in contrast to more recurrent cases, where this difference may be reduced or reversed.^22,55,56^ Other studies show greater amygdala volumes in individuals who are not currently depressed, but may be at risk for MDD due to elevated anxiety symptoms in the clinical^57^ or subclinical^58,59^ range, high trait negative affect,^60^ early-life stress,^61^ or positive family history of depression.^62^ Similarly, increased volume or thickness in various cortical regions have been reported in individuals with first-episode MDD,^63^ a positive family history of the disorder^62^ or comorbid anxiety.^64^ Although we did not find a difference in amygdala volume between individuals as a function of relatively higher or lower early life stress in the human sample, in partial agreement with this prior work, we did see a stress-related volumetric increase in occipital fusiform gyrus, a region implicated in emotion processing.^65^ Taken together, these findings suggest that the stress-induced volumetric changes we report may better reflect a phenotype of anxiety, depression vulnerability, or early stages of depression, rather than recurrent mood impairment. Further in line with this notion, in the rodent model we observed stronger effects in tests capturing anxiety-like behavior (e.g., EPM, NSF, time spent in the shelter zone), rather than other more depression-specific aspects of elevated emotionality such as anhedonia (e.g., cookie test, sucrose consumption) or helplessness-like behavior (forced swim test).

Notably and unlike the other brain regions with larger volumes in the UCMS group, the amygdala uniquely manifested a stress-related increase in number and strength of structural covariance connections to other network nodes. Despite substantial cross-species differences in brain parcellation schemes (155 vs. 56 ROIs), a strikingly similar increase in amygdala covariance emerged in the human sample. Structural covariance correlates partially with measures of both functional^66^ and structural^67^ connectivity and may reflect synchronized plastic changes occurring across multiple brain regions over time.^68^ Thus, the cross-species stress-related increase in amygdala strength and degree centrality may indicate an increased ability of the amygdala to ‘drive’ behavior through enhanced connections across an expanding subnetwork of behaviorally-relevant nodes. This notion is further supported by the observation that across species, the amygdala gained covariance with regions involved in the regulation of behavioral output, including threat avoidance and the fight-or-flight response. Notably, however, our findings highlight the increased centrality of the amygdala within a larger stress-altered structural covariance network. Our results are thus broadly consistent with classic work suggesting that chronic overactivation of the stress response, which is often observed in depression, may manifest on the neural circuit level as hyperfunctioning amygdala, possibly coupled with hypofunctioning in other key nodes of the corticolimbic circuit.^69^

Further suggestive of enhanced connectivity, the basolateral amygdala of UCMS-exposed mice showed higher density of PSD95, a scaffolding protein involved in neural plasticity and stabilization of long-term potentiation at excitatory synapses,^70^ consistent with previous reports of increased local synaptic number and strength following stress-exposure.^71,72^ The significant effect of chronic stress on amygdala PSD95 puncta density rather than VGLUT1 or MAP2 expression may be due to the specific cellular compartment assessed by each of these markers. The lack of significant changes in VGLUT1 and MAP2 density implies that basolateral amygdala afferent projections and dendritic arborization are affected in proportion to the volumetric expansion of the amygdala while the significant increase in PSD95 may reflect a dual stress-related effect on synaptic morphology and postsynaptic activity of the neurons in this brain region.

While the synaptic changes we observed are modest in size, they are within the range of classically reported spine density changes in rodent chronic stress studies^73–75^ (see ref. 51 for review). Indeed, these prior studies reported that UCMS induces a 25% increase post-synaptic density thickness, 20% increase in length of the synaptic active zone, but only 8–10% of number of spine/10 μm^2^ in the basolateral amygdala, the zone we analyze in our study. This is consistent with our findings. The small effect size and magnitude of effects of UCMS may explain why the correlation between PSD95 puncta density and behavior did not reach significance. This may also suggest that structural and synaptic changes in other brain regions can contribute to the expression of behavioral deficits induced by chronic stress.

In the human sample, the childhood trauma-related structural synchrony patterns may reflect enhanced maturational coupling between the amygdala and other regions, including those implicated in motivation (putamen, pallidum) and socioemotional processing (temporal fusiform gyrus). This altered coupling may developmentally bias brain function to enhance vulnerability to depression and anxiety in adulthood. As our UCMS model used adult mice, the cross-species structural covariance convergence we observe further suggests shared features between amygdala-centered trauma-related changes in maturational coupling and stress-induced plasticity in adulthood, although longitudinal studies in both mice and humans would be required to test this notion directly.

Although human imaging studies have associated current MDD with reduced amygdala functional coupling particularly with prefrontal regulatory regions,^10,11,76,77^ enhanced connectivity of the amygdala with these and other regions has been demonstrated following exposure to stress^78–80^ and trauma^10,81–83^ or in individuals with high anxiety.^10,59^ Notably, these prior studies relied on fMRI, which may be of more limited translational value, as it is state-dependent and relatively challenging to implement in preclinical work. While broadly consistent with these prior human fMRI findings, our results further identify increased amygdala structural covariance as a potential novel substrate of stress-related behavioral emotionality that may be more stable and conserved across species. In addition, we show that this relative strengthening of amygdala structural covariance occurs against the background of overall disrupted network-wide local clustering and reduced modularity. These concurrent findings suggest that the expansion of the amygdala as a network hub is not without tradeoff, including reduced integrity of other potentially behaviorally-relevant subnetworks. Thus, the network reorganization patterns associated with stress may reflect a highly conserved reallocation of neural network resources in response to a stress-induced shift in behavioral priorities. Furthermore, decreased network modularity suggests structural dedifferentiation, which may in turn make the brain over-specialized for a limited functional repertoire and detract from its ability to adapt to future changes in environmental priorities.^84^

Our study is not without limitations. First and foremost, we focused our analyses on brain volume and structural covariance as our sole imaging modalities. While ex vivo measures of brain structure in preclinical research have high translational potential due to their superior spatial resolution and relative ease of collection, future studies should employ multimodal characterization of stress-related changes in brain networks in vivo to complement the current findings with functional data. Second, our study focused on characterizing the behavioral, cellular and volumetric effects of one chronic stress paradigm in a single mouse strain and in males. While the direct extension of these results to a gender-balanced human sample strongly supports their generalizability, future preclinical studies in other mouse strains or other stress models in both males and females may yield additional insight into sex-specific pathways and the individual differences involved in the response to stress. Finally, in the human sample early childhood trauma was only assessed retrospectively via self-report in the absence of any extensive longitudinal clinical characterization. Future studies utilizing prospective longitudinal designs with repeated clinical assessments are likely to provide more fine-grained insight into how childhood trauma may translate into adult anxiety and/or depression.

Limitations notwithstanding, we provide initial translational evidence for convergent complex structural covariance network alterations associated with chronic stress in mice and early-life stress in humans. These findings and their associated methodology may thus represent a novel translational platform for the study of depression and anxiety and their shared stress-dependent risk pathways. Notably, the current results suggest that stress-related whole-brain and amygdala-specific structural covariance patterns may be more conserved across species than volumetric differences alone. As our observed behavioral and neural phenotypes likely capture early stages of mood and anxiety pathology or risk thereof, rather than more chronic forms of impairment, our findings may facilitate the development of methods for early identification of vulnerable individuals and targeted early intervention and prevention efforts in future translational work.

## Electronic supplementary material

All supplementary materials
