# Supplementary material for: Shifting priorities: highly conserved behavioral and brain network adaptations to chronic stress across species
Source: Transl Psychiatry. 2018 Jan 22;8:26. doi: 10.1038/s41398-017-0083-5 (PMC5802514; doi:10.1038/s41398-017-0083-5)
Supplement: Supplementary file 1 — All supplementary materials [file 41398_2017_83_MOESM1_ESM.doc]

**Supplemental Information for**

**Shifting Priorities: Highly conserved behavioral and brain network adaptations to chronic stress across species**

Yuliya S. Nikolova^1^§, Keith A. Misquitta^1,2^§, Brad R. Rocco^1^, Thomas D. Prevot^1^, Annchen R. Knodt,^3^ Jacob Ellegood^4,5^, Aristotle N. Voineskos,^1,6^ Jason P. Lerch^4,5^, Ahmad R. Hariri^3^, Etienne Sibille^1,2,6^, Mounira Banasr^1,2,6,*^.

**Table content**

**Supplementary Methods (p2-13)**

**Supplementary Figure 1 (p14)**

**Supplementary Figure 2 (p15)**

**Supplementary Figure 3 (p16)**

**Supplementary Figure 4 (p17)**

**Supplementary Table 1 (p18-19)**

**Supplementary Table 2 (p20-23)**

**Supplementary Table 3 (p24-26)**

**Supplementary Table 4 (p27-34)**

**Supplementary Table 5 (p35)**

**Supplementary Table 6 (p36-38)**

**Supplementary Table 7 (p39-41)**

**Supplementary References (p42-45)**

**Supplementary Methods**

**Mouse Sample**

*Animals:* Eight week-old Balb/c mice (Charles River Laboratories, Quebec, Canada) were housed under normal conditions, with *ad libitum* access to food, water and a 12/12 light/dark cycle except when specified for UCMS paradigm and for behavioral testing. All animal procedures were performed in accordance with the Canadian Council on Animal Care (CCAC) guidelines.

*UCMS:* Mice were exposed to UCMS for 5 weeks (3-4 randomized stressors/day) as in^1^ (n=12/group). Stressors included: forced bath, wet bedding, aversive smell (fox or bobcat urine), light cycle reversal or disruption, tilted cage, reduced space, restraint, bedding change, used bedding, no bedding or nestlet removal. In addition, UCMS mice were single housed while controls were group housed (~3-4 per cage). Control animals were only handled for weight and coat state and behavioral assessment. Except for coat state assessment, mice were not subjected to UCMS on days of behavioral testing.

**Human Sample**

*Participants:* One thousand, two-hundred and eight participants from the Duke Neurogenetics Study (DNS, 703 women, mean age 19.68±1.26) had valid structural MRI and Childhood Trauma Questionnaire (CTQ^2^). The CTQ assesses the severity of five types of trauma: emotional abuse, physical abuse, emotional neglect, physical neglect, and sexual abuse. We selected for analysis participants scoring in the top 25% (>37, n=299, 177 women) and bottom 25% (<27, n=237, 143 women) of the observed range of total CTQ scores. All Duke Neurogenetics Study (DNS) participants provided informed consent in accordance with Duke University guidelines. Current depressive and anxiety symptomatology was measured using the Mood and Anxiety Symptom Questionnaire (MASQ^3^) General Distress Depression and Anxiety scales, respectively. Current or past major depressive episodes were assessed with the electronic Mini International Neuropsychiatric Interview.^4^ For full exclusion criteria, see Nikolova et al.^5^

**Mouse Behavioural Testing**

*Coat quality assessment:* Coat state was assessed weekly for 5 weeks on 7 anatomical regions (head, neck, dorsal/ventral coat, forepaw, hindpaw and tail) of the mouse’s body by attributing a score of 0, 0.5, or 1 for each area from maintained to unkempt.

*Home-cage-like behavioral assessment:* To assay mouse regular behavior, we used the Noldus PhenoTyper apparatus (Leesburg VA, USA) an observation cage designed for video-tracking and software EthoVision 10. This apparatus includes an integrated infrared sensitive camera that allows for measuring for each mouse the time spent in 3 predefined zones (food, drinking and shelter zones) during the dark cycle. We also designed a test that measured the animal’s response to an anxiogenic stimulus or challenge (white LED spotlight) over the food zone. Baseline behavior correspond to the average time spend in each zone for the four first hours into the dark cycle (7:00pm to 11:00pm). Then, the anxiogenic white spotlight challenge was applied for 1hr from 11:00pm to 12:00am over the food zone. Time spent in each zone is measured hourly, every week (day 0, 7, 14, 21, 28, 34). Analysis of the group differences focused on weekly average baseline behavior as well as the response to challenge (comparing before, during and after the challenge differences).

*Classical Behavioral Tests:* After 5 weeks, UCMS and control mice were tested in multiple tests assessing anxiety-like and depressive-like behaviors every other day as in Soumier et al ^1^. Mice subjected to UCMS mice continued receiving stressors on “non-testing” days. The order of the tests was chosen to minimize interactions between tests (i.e elevated plus maze and open field before forced swim test (FST) or sucrose consumption before cookie test).

*Elevated plus maze:* On day 36, mice are individually placed in an elevated maze (55cm from the floor) containing four white plexiglass arms (90 degree), two open arms (27x5 cm) and two closed arms (27x5x15 cm). Mice are allotted a 5min exploration period in a dimly illuminated room (~28 lux). Sessions were videotaped and analysed using ANY-Maze tracking system (Stoeling, IL). Number of entries and time in the closed and open arms were measured.

*Open Field test:* On day 38, mice were tested in an open arena (~70x70cm width) for 20min. Time spent and number of entries in the center zone as well as total distance traveled were measured using ANY-Maze.

*Forced Swim Test:* On day 40, mice are placed in a transparent tank filled with water (25cm, 23-24^o^C) for 10 min. A manual count of time spent immobile was performed by an experimentor blinded to the animal treatment history.

*Novelty suppressed feeding test:* After overnight food deprivation (~14-16 hours), mice were placed in a novel arena (45 x 30 x 27 cm) with standard food pellets in the middle, under dim light (28-30 lux). Latencies to approach and to feed on the pellet (in seconds) were measured during the 12-min test. Latency to feed on a food pellet within the animal’s home cage served as control for experimental bias due to appetite drive.

*Sucrose consumption test:* Mice were habituated for 72hrs to a solution of 1% sucrose. Following overnight (~14 hours) fluid deprivation, total sucrose intake (g) for each mouse was measured for 1hr. Sucrose bottles were then switched to water. Water intake was measured (g) 1hr-long test following overnight fluid deprivation the next day to control for total fluid intake.

*Cookie Test:* Mice were tested in an apparatus (60 x 38 cm) containing a clear transparent chamber divided into 3 aligned sections (20 x 38 cm) with different wall colors (white, gray, and black). Each of the compartments are linked together by rectangular holes in each wall. After habitution to a small piece of Oreo® cookie (quarter of the cookie, no cream filling) for 5 days, mice were placed in the apparatus in the white compartment and the cookie was placed in the center of black chamber. The mouse latency to approach and eat the cookie was measured for an alloted 12-min period for 2 consecutive days.

**Mouse Brain Tissue Preparation**

On day 44, the mice were anesthetized with avertine (125mg/kg, i.p.) and intracardially perfused with 30mL of 0.1M PBS containing 10U/mL heparin and 2mM ProHance (a Gadolinium contrast agent) followed by 30mL of 4% paraformaldehyde (PFA) containing 2mM ProHance.^6, 7^ Perfusions were performed with a Pharmacia minipump at a rate of approximately 100mL/hr. After perfusion, mice were decapitated and the skin, lower jaw, ears, and the cartilaginous nose tip were removed. The brain and remaining skull structures were post-fixed in 4% PFA + 2mM ProHance overnight at 4^o^C then transferred to 0.1M PBS containing 2mM ProHance and 0.02% sodium azide for at least 7 days prior to MRI scanning.

**Mouse MRI Data Acquisition and Preprocessing**

A multi-channel 7.0 Tesla MRI scanner (Varian Inc., Palo Alto, CA) was used to image the brains within skulls. Sixteen custom-built solenoid coils was used to image the brains in parallel.^8^ We acquired a T2- weighted, 3-D fast spin-echo sequence, with a cylindrical acquisition of k-space,^9^ and with a TR (repetition time) of 350 ms, and TEs (Echo time) of 12 ms per echo for 6 echoes, field-of-view of 20 x 20 x 25 mm3 and matrix size = 504 x 504 x 630 giving an image with 0.040 mm isotropic voxels. Total imaging time was 14 h for 30 brains. Three control and one UCMS animals were removed from analysis due to brain damage at removal (n=1) or brain defects such as non-crossing corpus callosum fibers (n=2), giving a final sample of n=9 control and n=12 UCMS mice with usable MRI data.

To visualize and compare any changes in the mouse brains, the images are linearly (6 parameters followed by a 12 parameters) and non-linearly registered together. All registrations were performed with a combination of mni_autoreg tools^10^ and advanced normalization tools (ANTs).^11, 12^ All scans are then resampled with the appropriate transform and averaged to create a population atlas representing the average anatomy of the study sample. The result of the registration is to have all scans deformed into alignment with each other in an unbiased fashion. This allows for the analysis of the deformations needed to take each individual mouse’s anatomy into this final atlas space, the goal being to model how the deformation fields relate to treatment.^7, 13^ The jacobian determinants of the deformation fields are then calculated as measures of volume at each voxel for each brain region. Significant volume changes can then be calculated by warping a pre-existing classified MRI atlas onto the population atlas, which allows for the volume of 159 segmented structures including cortical lobes, large white matter structures (i.e. corpus callosum), ventricles, cerebellum, brain stem, and olfactory bulbs^14-16^ to be assessed in all brains. While in vivo functional imaging is feasible in rodents,17 we opted for ex vivo structural imaging, in order to capitalize on the superior spatial resolution afforded by longer scanning times in ex vivo protocols (40 micron isotropic voxels vs. ~150 microns in vivo) and preclude any confounding effects of a stressful in vivo scanning session.

Given limited evidence of rodent-human convergence in hemispheric lateralization of emotion processing and expression,^18, 19^ all our MRI analyses focused on ROI volumes averaged across hemispheres. For each animal, the absolute volume (in mm^3^) of each brain region was divided by the total brain volume prior to analysis. This normalization step was performed to account for inter-individual variability in absolute head and body size unrelated to the stress manipulation, as well as variability in absolute volume attributable to normal variability in the execution of our manual perfusion protocol. Given the lack of evidence of cross-species convergence of hemispheric specialization, all our MRI analyses focused on ROI volumes averaged across hemispheres. All regional volumes were normalized by total brain volume for each animal to account for inter-individual variability in absolute head and body size unrelated to the stress manipulation, as well as variability in absolute volume attributable to the manual perfusion protocol.

**Fluorescence Immunohistochemistry and Confocal Microscopy Analysis**

Following MR imaging, brains were removed from the skull and then rinsed in a graded series of sucrose changes for cryoprotection. Tissue sections (14 µm-thickness) containing the basolateral amygdala were sectioned on a cryostat, immediately mounted on glass slides, and stored at -20°C until processed for immunohistochemistry. One animal was removed from the analysis due amygdala damage at cryosectioning. Two sections per animal were then processed for fluorescence immunohistochemistry. Sections were rinsed in 1X phosphate-buffered saline (PBS, 20min) at room temperature (RT), incubated in 0.01M sodium citrate in distilled H2O at 80°C for 15 minutes. Sections were then incubated in 0.3% Triton X-100 in PBS for 20 minutes, followed by 1h (RT) in 20% donkey serum (0.3% PBS-Triton), and then incubated at 4°C in PBS containing 2% donkey serum and primary antibodies specific for postsynaptic density protein 95 (PSD95; rabbit host, 1:100, Cell Signaling Technology, Danvers, MA, product #2507, lot 2), microtubule-associated protein 2 (MAP2; mouse host, 1:1000, Sigma-Aldrich, St. Louis, MO, product #M9942, lot 024M4836V), and vesicular glutamate transporter 1 (VGlut1; guinea pig host, 1:500, Synaptic Systems, Goettingen, Germany, product #135304, lot 135304/31) overnight. Sections were then rinsed and incubated in 2% donkey serum and secondary antibodies (2h, donkey host, 1:500 for all) conjugated to Cy3 (Jackson ImmunoResearch Inc., West Grove, PA), Alexa 488 (Jackson ImmunoResearch Inc., West Grove, PA), and CF405M (Biotium, Hayward, CA), which were used to detect PSD95, MAP2, and VGLUT1 immunoreactivity, respectively. The sections were then mounted and coverslipped (Vectashield Antifade Mounting Media, Vector Laboratories, Burlingame, CA), and stored at 4°C until imaged.

MAP2 immunoreactivity was used to delineate the boundaries of the basolateral amygdala. Three to 5 systematic randomly sampled image stacks were taken within each amygdala using a sampling grid of 180 x 180 µm2. An investigator blinded to subject and experimental group collected an average of 16.3 (± 3.7) image stacks per animal (both hemispheres). Data were collected on an Olympus IX83 inverted microscope equipped with a spinning disk confocal unit and Hamamatsu Orca-Flash4.0 V2 digital CMOS camera using a 60X 1.4 NA SC oil immersion objective. The equipment was controlled by SlideBook 6 (Intelligent Imaging Innovations, Denver, CO); the same software package was used for post-image processing. Three dimensional image stacks (intervals separated by 0.25 µm in the z-dimension) were acquired over 25% of the total tissue thickness determined by the degree of antibody penetration and fluorescence detectability. The stacks were collected using the same exposure settings per each fluorescent channel and then deconvolved using the AutoQuant adaptive blind deconvolution algorithm (Media Cybernetics, Inc., Rockville, MD). All data segmentation was done using MATLAB (R2016). For data segmentation of the MAP2 channel, the Ridler-Calvard iterative thresholding algorithm^20^ was used and all objects ≥ 0.05 µm3 were analyzed. Data segmentation of the PSD95 and VGLUT1 channels was performed as described.^21^

*Human MRI Acquisition and Preprocessing*

Participants were scanned on one of two identical research-dedicated GE MR750 3T scanners at the Duke-UNC Brain Imaging and Analysis Center. Each scanner was equipped with high-power high-duty cycle 50-mT/m gradients at 200 T/m/s slew rate and an eight-channel head coil for parallel imaging at high bandwidth up to 1 MHz. We obtained T1-weighted images using a 3D Ax FSPGR BRAVO sequence (TR=8.148 s; TE=3.22 ms; 162 axial slices; flip angle, 12°; FOV, 240 mm; matrix=256 × 256; 1 mm slice thickness with no gap) for a total scan time of 4 min and 13 s. Regional gray matter volumes were determined using the unified segmentation^22^ and DARTEL normalization^23^ modules in SPM12 (http://www.fil.ion.ucl.ac.uk/spm). Using this approach, individual T1-weighted images were segmented into gray, white, and CSF images then non-linearly registered to the existing IXI template of 550 healthy subjects averaged in standard Montreal Neurological Institute space, available with VBM8 (http://dbm.neuro.uni-jena.de/vbm/). Subsequently, gray matter images were modulated for nonlinear effects of the high-dimensional normalization to preserve the total amount of signal from each region, and smoothed with an 8mm FWHM Gaussian kernel. The voxel size of processed images was 1.5×1.5×1.5 mm. Following preprocessing, gray matter volumes were extracted for each cortical and subcortical region as delineated in the Harvard-Oxford Atlas (<http://fsl.fmrib.ox.ac.uk/fsl/fslwiki/Atlases>), by summing across the voxels within each region bilaterally, and then averaging across hemispheres. We used the Harvard Oxford Atlas^24, 25^ to parcellate the brain into 56 cortical and subcortical gray matter ROIs per hemisphere. To keep the analysis as similar as possible to the one carried out in the mouse model, individual ROI volumes were averaged between hemispheres and additionally residualized for sex and total brain volume.

**Statistical Analysis**

*Mouse behavior and cellular analysis:* Weight, coat state and performance in the phenotypers were measured weekly and data were analyzed using repeated measures ANOVA (Statview software), followed by Bonferroni-corrected *post-hoc* comparisons. Data in the classical behavioral tests were analyzed using a t-test. In order to normalize the data across multiple tests a z-score methodology was applied.^26^ Z-normalization allows for assessing multiple behavioural outcomes into one emotionality dimension. The z-score describes if the amount of standard deviation of a measure is above or below the mean of the control group. The average of the z-scores of each test was calculated. Significance was set at 5%. All data are presented as mean ± SEM. In addition, to create summary scores capturing behavioral emotionality dimensionally across groups, a principal component analysis (PCA) was conducted on 34 major behavioural variables (see **Supplementary Figure. S3A**). For longitudinal measures, the PCA included the first and last assessment only. An independent samples t-test was used to assess differences in the density and fluorescence intensity levels of PSD95- and VGLUT1-immunoreactive puncta, and the volume and fluorescence intensity levels of MAP2-immunoreactive objects between groups.

Mouse and human volumetric MRI analysis: The volume of each ROIs was compared between the UCMS-exposed and the control groups, using a general linear model with an overall false discovery rate (FDR) of q<0.05. In the mouse, additional regression analyses examined the association between volume in each ROI and behavioral PC1.

Cross-species structural covariance network analysis: Correlation matrices were created separately for the stress and control groups using 155 ROIs (i.e., removing the 4 ventricles) in the mouse and all 56 ROIs in the human sample. Consistent with prior work,^27^ negative correlations were discarded and correlation matrices were thresholded at a broad range of density thresholds (0.10-0.30), sequentially considering the top 10-30% strongest connections in increments of 1%. Each thresholded matrix was converted into a weighted graph, where each node represented the volume of an ROI and each edge represented the interregional correlation in brain volumes.

Using the ‘igraph’ package for R,^28^ at each density threshold the following network-level measures were computed separately for the control and stress groups: 1) transitivity, global clustering coefficient & modularity (based on the Girvan-Newman algorithm^29^), reflecting different aspects of network segregation and capacity for specialized local processing; and 2) average path length, indicative of global integration capacity of the network. For each ROI showing an effect of stress in our univariate volumetric analysis, we computed the following node-level metrics to assess changes in its position or “hubness” in the network: 1) degree centrality (i.e., number of connections to other nodes); 2) strength (the weight of all present connections normalized by the mean node-level network strength); 3) betweenness & closeness centrality (normalized by the average node-level centrality of the network; only computed for nodes and density ranges with significant degree centrality differences).

For each threshold and metric, permutation testing (n=10,000) was performed to obtain a null distribution, against which empirical two-tailed p-values were computed. A between-group parameter difference was considered significant if 1) the direction of effect was consistent across the vast majority (>90%) of density thresholds tested,^27^ and 2) its empirical p value reached p<0.05 for at least two consecutive thresholds. Results were considered suggestive if they met criterion 1) and were trending for at least 3 consecutive density thresholds. We did not require that results be significant in the same density range across species, as the standard parcellation schemes used were very different across mouse and human (i.e., 155 vs 56 ROIs, respectively). Furthermore for all results considered significant or suggestive at specific density ranges as outlined above, aggregate significance across all density thresholds was confirmed using area under the curve (AUC) analysis.^30^ Finally, between-group differences in these same measures were correlated with between-group differences in behavioral or self-reported emotionality (in mouse and human, respectively) across 10,000 permutations (**Supplementary Figure. S4**).

Networks were visualized using Cytoscape^31^ at the lowest density where the majority of between-group effects emerged. Whole networks were visualized using the Attribute Circle Layout organized by degree, with degree increasing clockwise and the highest-degree node always placed on top of the circle. Specific subnetworks of interest were visualized using the Edge-Weighted Spring Embedded Layout option in Cytoscape, which is based on the “force-directed” layout algorithm originally implemented by Kamada & Kawai (1989).^32^

**Code Availability**

The computer code used to generate the network results is available upon request.

**Supplementary Figures**

**
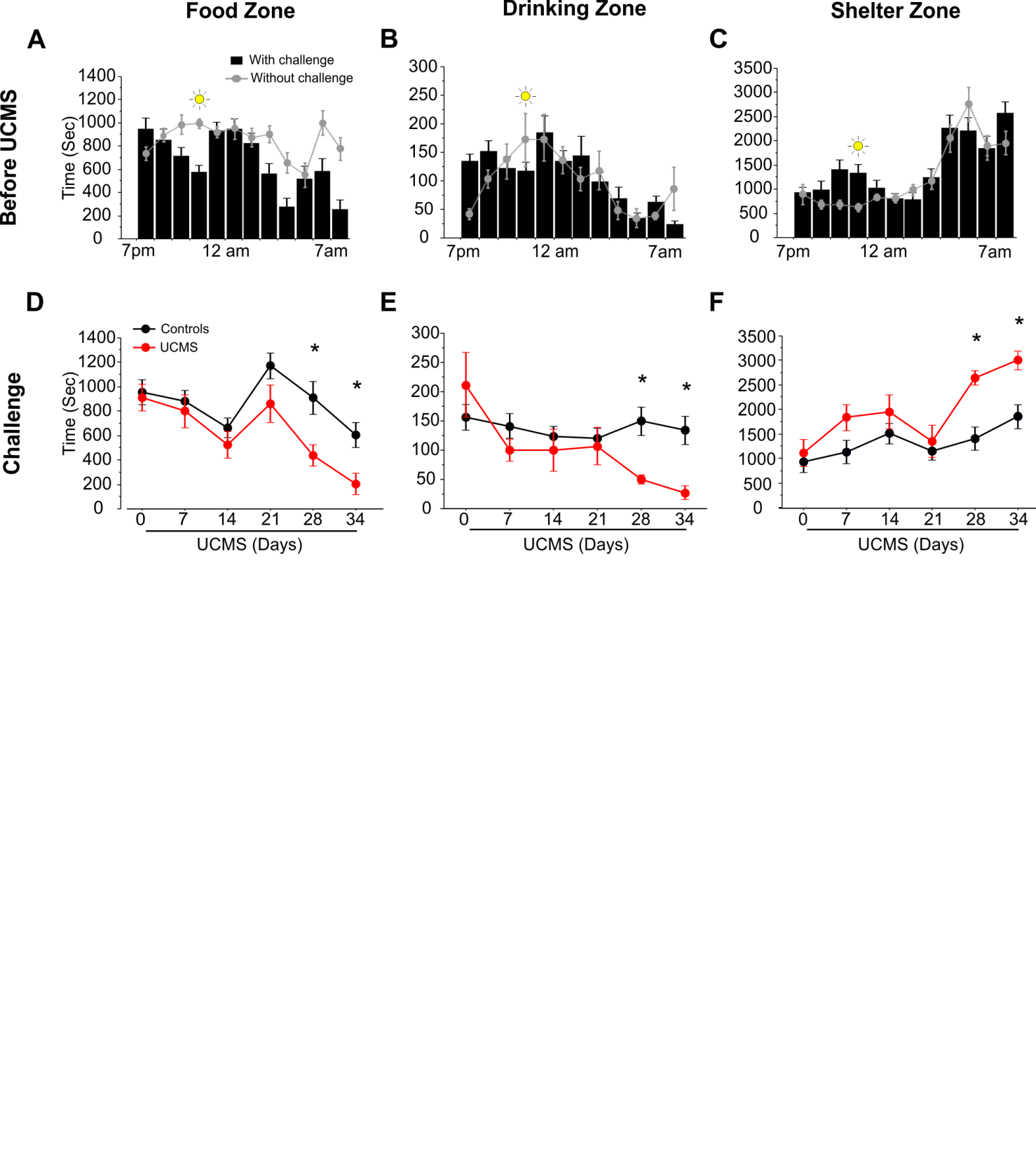
**

**Supplementary Figure 1. Spotlight challenge alters baseline behavior in both control and UCMS mice. (A-C) Spotlight challenge alters home cage-like behavior.** Hourly monitoring of home cage-like behavior revealed that in control conditions mice spend more time in the food **(A)** and drinking **(B)** zones earlier in the dark cycle, and greater time in the shelter zone **(C)** later in the cycle (gray curve). This home cage-like behavior is altered by the application of a spotlight over the food zone (11pm to 12am), where animals avoid the zone (black histogram). **(D-F) Spotlight challenge throughout the UCMS exposure.** Repeated measure ANOVA revealed significant effect of stress and/or time in all three zones on the weekly monitoring of the animals during the challenge. UCMS-exposed mice show a significant decrease in the amount of time spent in the food zone **(D)** (main effect stress: F_1,22_=10.33, p<0.01; time: F_5,110_=9.234, p<0.001; no interaction) and drinking zone **(E)** (interaction stress x time: F_5,110_= 3.021; p<0.05) while showing an increase in the amount of time spent in the shelter zone **(F)** (main effect stress: F_1,22_=11.350; p<0.01; time: F_5,110_; 10.724, p<0.0001) (Post hoc analysis *p<0.05 compared to home cage controls).


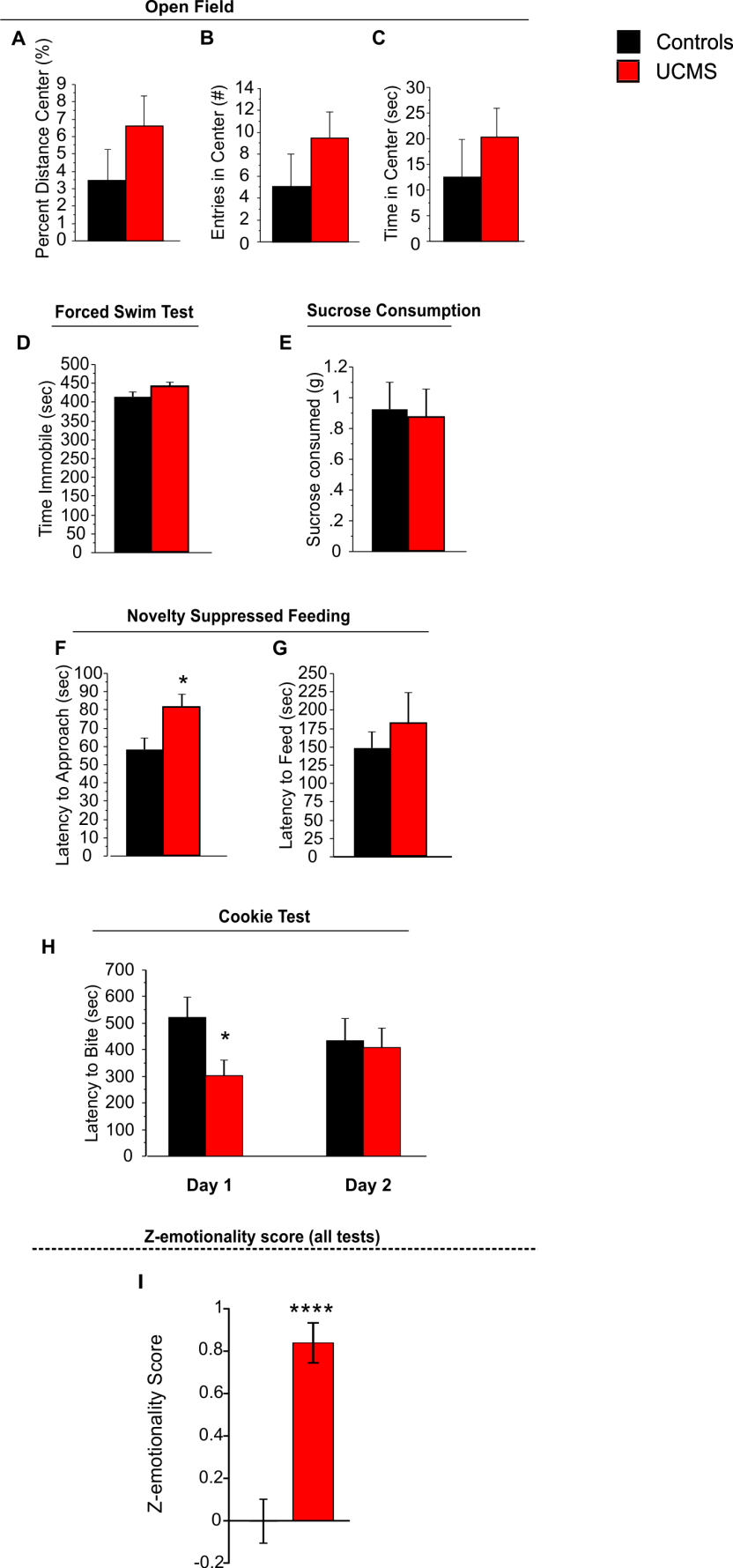
**Supplementary Figure 2. UCMS has mixed effects on classical behavior but overall increases behavioral emotionality.** In the open field, UCMS-exposed mice showed no significant change in distance travelled in the center **(A)**, entries in the center **(B)** or time spent in the center **(C)**. **(D)** No significance between groups was found in time spent immobile in the forced swim test or **(E)** in sucrose intake in the sucrose consumption test. In the novelty suppressed feeding test, UCMS-exposed mice showed a significant increase in the latency to approach **(F)** (*t test*, * p<0.05) but no significant change in the latency to feed **(G)**. In the cookie test **(H)**, UCMS induced a decrease in the latency to bite on the first test day but not change on the second day of testing (*p<0.05). **(I)** Overall UCMS induced an increase in emotionality index by the z-score calculated from all behavioral tests measuring emotion-like behavior, including phenotyper, elevated plus maze, open field, forced swim, sucrose consumption, novelty suppressed feeding and cookie tests (*p<0.0001).


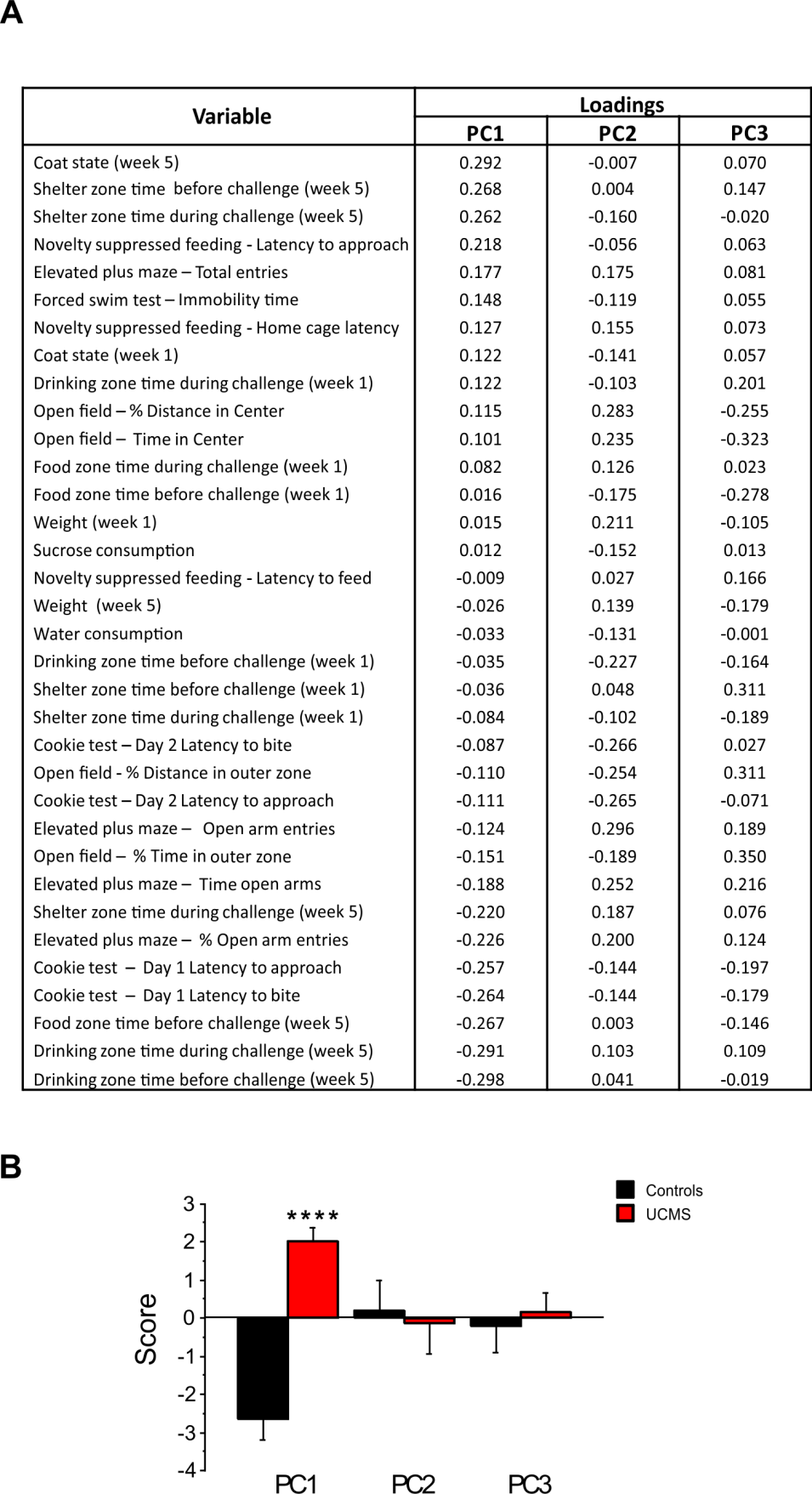
**Supplementary Figure 3.** **Results from a principal component analysis (PCA) on behavioral data across measures. (A)** Raw variable loadings onto the top three principal components. Variables are sorted by PC1 loadings. **(B)** Between-group (Control vs. Stress) differences in PC1, PC2 and PC3 (**** p<0.0001).


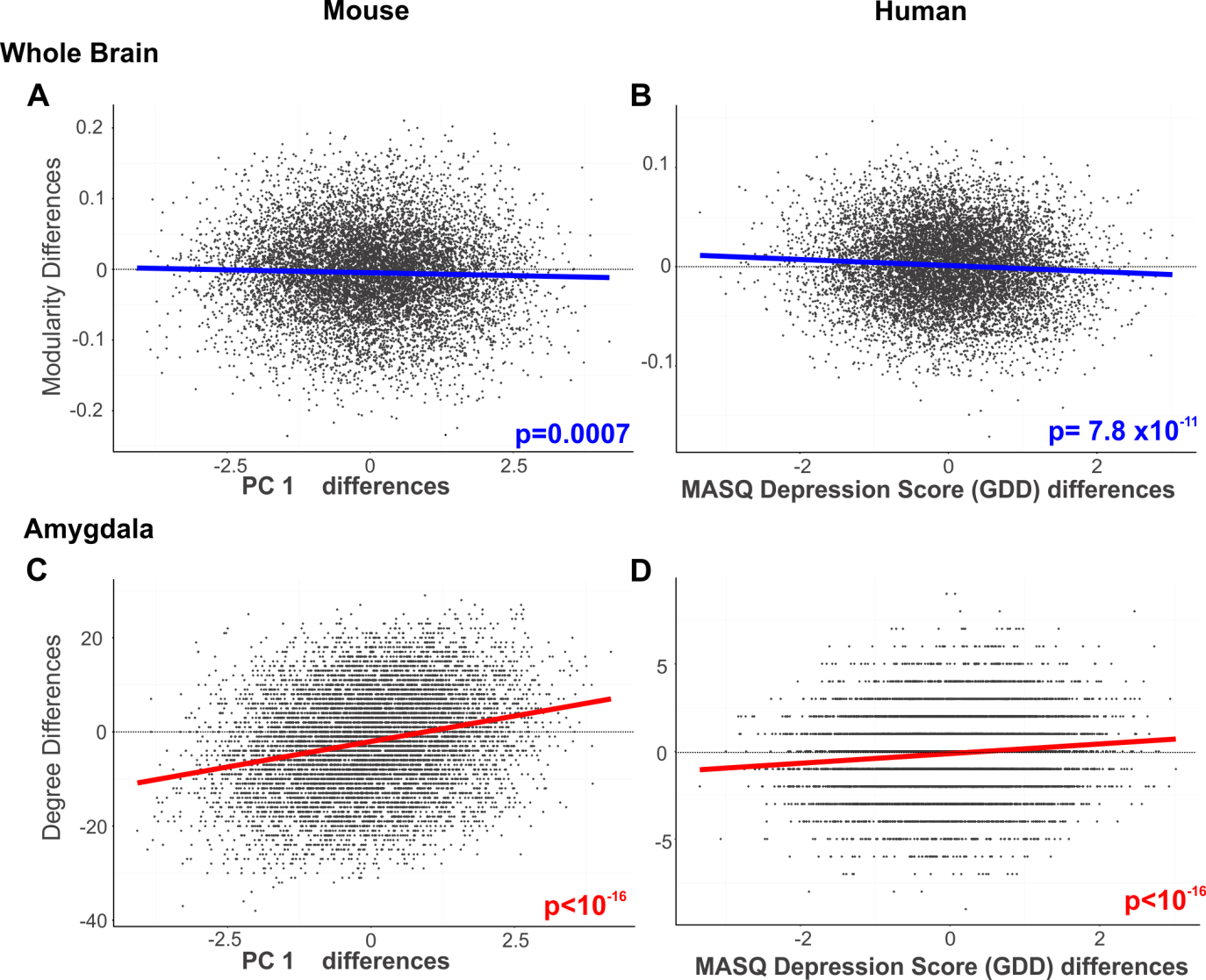


**Supplementary Figure 4.** **Between-group differences in network and node attributes were proportional to between-group differences in emotionality.** Across 10,000 permutations, between-group differences in network modularity were negatively correlated with those in behavioral PC1 in the mouse sample **(A)**, and the Mood and Anxiety Symptom Questionnaire General Distress Depression (MASQ GDD) scale in the human sample **(B)** at 22% and 12% density, respectively. Between-group differences in amygdala degree were positively correlated with those in PC1 **(C)** and MASQ GDD **(D).** All p values < 0.001.

**Supplementary Table 1: Between-group (control vs. stress) volumetric comparison results for the 26 pre-selected regions of interest in the mouse sample, listed by significance (from smallest to largest uncorrected p value).**

| **Brain Region** | **Direction of Change** | **Group Effects** | | | **PC1 Effects** | |
| --- | --- | --- | --- | --- | --- | --- |
|  |  | **Variance Accounted For** | ***p*** | ***q*** | **Variance Accounted For** | ***p*** |
| **Cingulate Cortex: Area 32** | **↑** | **39.01%** | **0.002** | **0.041** | **26.87%** | **0.0161** |
| **Medial Orbital Cortex** | **↑** | **34.88%** | **0.005** | **0.041** | **23.24%** | **0.0269** |
| **Frontal Association Cortex** | **↑** | **34.13%** | **0.005** | **0.041** | **19.61%** | **0.0444** |
| Dorsolateral Orbital Cortex | ↑ | 33.23% | 0.006 | 0.041 | 18.12% | 0.0544 |
| **Amygdala** | **↑** | **30.52%** | **0.009** | **0.049** | **22.18%** | **0.0312** |
| Lateral Orbital Cortex | ↑ | 28.87% | 0.012 | 0.052 | 18.86% | 0.0492 |
| Nucleus Accumbens | ↑ | 24.65% | 0.022 | 0.082 | 15.24% | 0.0802 |
| Cingulate Cortex: Area 24B | ↑ | 20.65% | 0.038 | 0.125 | 18.87% | 0.0491 |
| Striatum | ↑ | 18.96% | 0.048 | 0.126 | 5.71% | 0.297 |
| Hippocampus | ↑ | 18.95% | 0.049 | 0.126 | 19.91% | 0.0426 |
| Ventral Orbital Cortex | ↑ | 17.67% | 0.058 | 0.136 | 12.70% | 0.1128 |
| Cingulate Cortex: Area 25 | ↑ | 16.12% | 0.071 | 0.136 | 8.52% | 0.1991 |
| Globus Pallidus | ↑ | 16.02% | 0.072 | 0.136 | 9.15% | 0.1826 |
| Hypothalamus | ↑ | 15.90% | 0.073 | 0.136 | 17.86% | 0.0563 |
| Cingulate Cortex: Area 29A | ↓ | 11.15% | 0.139 | 0.241 | 11.26% | 0.1371 |
| Insular Region: Not Subdivided | ↑ | 10.48% | 0.152 | 0.241 | 15.69% | 0.0755 |
| Cingulate Cortex: Area 24A | ↑ | 10.23% | 0.158 | 0.241 | 8.88% | 0.1894 |
| Lateral Parietal Association Cortex | ↑ | 4.92% | 0.334 | 0.459 | 0.67% | 0.7251 |
| Dentate Gyrus Of Hippocampus | ↑ | 4.89% | 0.335 | 0.459 | 11.71% | 0.1289 |
| Cingulate Cortex: Area 29B | ↓ | 3.93% | 0.389 | 0.506 | 0.48% | 0.7655 |
| Medial Parietal Association Cortex | ↑ | 3.54% | 0.414 | 0.513 | 0.00% | 0.9844 |
| Thalamus | ↓ | 1.80% | 0.562 | 0.664 | 3.51% | 0.4162 |
| Cingulate Cortex: Area 29C | ↑ | 1.01% | 0.665 | 0.752 | 0.00% | 0.9926 |
| Midbrain | ↑ | 0.54% | 0.751 | 0.814 | 0.77% | 0.7059 |
| Temporal Association Area | ↓ | 0.14% | 0.874 | 0.909 | 7.05% | 0.2448 |
| Cingulate Cortex: Area 30 | ↑ | 0.01% | 0.962 | 0.962 | 0.11% | 0.8859 |

Note: Regions highlighted in bold font show both an effect of Group at the q<0.05 significance level and a significant correlation with PC1 (p<0.05 uncorrected).

**Supplementary Table 2:** Whole-brain between-group (control vs. stress) volumetric comparison results in the mouse sample, listed by significance (from smallest to largest uncorrected p value).

| **Brain Region** |  | **Group Effects** | | |
| --- | --- | --- | --- | --- |
|  | **Direction of Change** | **Variance Accounted For** | **p** | **q** |
| Lobule 10: Nodulus | ↓ | 45.27% | 0.001 | 0.092 |
| Lobule 10 White Matter | ↓ | 43.43% | 0.002 | 0.092 |
| Cingulate Cortex: Area 32 | ↑ | 39.01% | 0.003 | 0.110 |
| Facial Nerve: Cranial Nerve 7 | ↓ | 35.89% | 0.004 | 0.110 |
| Medial Orbital Cortex | ↑ | 34.88% | 0.006 | 0.110 |
| Cerebellar Peduncle: Middle | ↓ | 34.58% | 0.008 | 0.110 |
| Frontal Association Cortex | ↑ | 34.13% | 0.011 | 0.110 |
| Crus 1: Ansiform Lobule: Lobule 6 | ↓ | 33.92% | 0.012 | 0.110 |
| Dorsolateral Orbital Cortex | ↑ | 33.23% | 0.013 | 0.110 |
| Amygdala | ↑ | 30.52% | 0.014 | 0.141 |
| Claustrum | ↑ | 29.83% | 0.018 | 0.141 |
| Medulla | ↓ | 29.71% | 0.021 | 0.141 |
| Lateral Orbital Cortex | ↑ | 28.87% | 0.025 | 0.145 |
| Cerebral Peduncle | ↓ | 27.97% | 0.026 | 0.145 |
| Cerebellar Peduncle: Inferior | ↓ | 27.96% | 0.028 | 0.145 |
| Copula: Pyramis: Lobule 8 | ↓ | 25.24% | 0.029 | 0.179 |
| Dentate Nucleus | ↓ | 24.98% | 0.036 | 0.179 |
| Nucleus Accumbens | ↑ | 24.65% | 0.038 | 0.179 |
| Pontine Nucleus | ↓ | 24.31% | 0.046 | 0.179 |
| Amygdalopiriform Transition Area | ↑ | 24.18% | 0.047 | 0.179 |
| Dorsal Nucleus Of The Endopiriform | ↑ | 23.86% | 0.047 | 0.179 |
| Trunk Of Arbor Vita | ↓ | 23.62% | 0.048 | 0.179 |
| Posteromedial Cortical Amygdaloid Area | ↑ | 23.51% | 0.049 | 0.179 |
| Cuneate Nucleus | ↓ | 22.58% | 0.049 | 0.184 |
| Ventral Intermediate Entorhinal Cortex | ↑ | 22.57% | 0.050 | 0.184 |
| Dorsal Intermediate Entorhinal Cortex | ↑ | 22.44% | 0.051 | 0.184 |
| Ventral Nucleus Of The Endopiriform Claustrum | ↑ | 21.98% | 0.052 | 0.189 |
| Medial Septum | ↑ | 21.68% | 0.055 | 0.190 |
| Cingulate Cortex: Area 24B | ↑ | 20.65% | 0.056 | 0.211 |
| Striatum | ↑ | 18.96% | 0.060 | 0.249 |
| Hippocampus | ↑ | 18.95% | 0.065 | 0.249 |
| Pons | ↓ | 18.03% | 0.072 | 0.264 |
| Subependymale Zone: Rhinocele | ↑ | 17.87% | 0.075 | 0.264 |
| Ventral Orbital Cortex | ↑ | 17.67% | 0.075 | 0.264 |
| Lateral Ventricle | ↑ | 17.63% | 0.089 | 0.264 |
| Trunk Of Crus 2 And Paramedian White Matter | ↓ | 16.41% | 0.091 | 0.292 |
| Interpedunclar Nucleus | ↓ | 16.26% | 0.095 | 0.292 |
| Cingulate Cortex: Area 25 | ↑ | 16.12% | 0.097 | 0.292 |
| Globus Pallidus | ↑ | 16.02% | 0.103 | 0.292 |
| Hypothalamus | ↑ | 15.90% | 0.108 | 0.292 |
| Fourth Ventricle | ↓ | 15.54% | 0.108 | 0.298 |
| Lateral Septum | ↑ | 14.90% | 0.109 | 0.313 |
| Bed Nucleus Of Stria Terminalis | ↑ | 14.84% | 0.111 | 0.313 |
| Habenular Commissure | ↓ | 14.56% | 0.118 | 0.316 |
| Basal Forebrain | ↑ | 14.42% | 0.128 | 0.316 |
| Secondary Motor Cortex | ↑ | 13.84% | 0.130 | 0.335 |
| Simple Lobule: Lobule 6 | ↓ | 13.60% | 0.141 | 0.338 |
| Fastigial Nucleus | ↓ | 12.93% | 0.147 | 0.359 |
| Optic Tract | ↓ | 12.85% | 0.148 | 0.359 |
| Posterior Commissure | ↓ | 11.92% | 0.165 | 0.378 |
| Piriform Cortex | ↑ | 11.74% | 0.168 | 0.378 |
| Ventral Tenia Tecta | ↑ | 11.63% | 0.171 | 0.378 |
| Paraflocculus White Matter | ↓ | 11.46% | 0.174 | 0.378 |
| Lobule 9 White Matter | ↓ | 11.17% | 0.185 | 0.378 |
| Cingulate Cortex: Area 29A | ↓ | 11.15% | 0.194 | 0.378 |
| Dorsal Tenia Tecta | ↑ | 11.12% | 0.201 | 0.378 |
| Lobule 7: Tuber: Or Folium | ↓ | 11.09% | 0.206 | 0.378 |
| Crus 1 White Matter | ↓ | 10.93% | 0.218 | 0.378 |
| Corticospinal Tract Pyramids | ↓ | 10.77% | 0.221 | 0.378 |
| Claustrum: Ventral Part | ↑ | 10.76% | 0.226 | 0.378 |
| Nucleus Interpositus | ↓ | 10.72% | 0.227 | 0.378 |
| Copula White Matter | ↓ | 10.53% | 0.227 | 0.378 |
| Anterior Commissure: Pars Posterior | ↑ | 10.48% | 0.236 | 0.378 |
| Insular Region: Not Subdivided | ↑ | 10.48% | 0.252 | 0.378 |
| Cingulate Cortex: Area 24A | ↑ | 10.23% | 0.263 | 0.386 |
| Paraflocculus: Pfl | ↓ | 10.08% | 0.267 | 0.387 |
| Superior Olivary Complex | ↓ | 9.97% | 0.273 | 0.387 |
| Posterolateral Cortical Amygdaloid Area | ↑ | 9.47% | 0.274 | 0.409 |
| Stria Medullaris | ↓ | 9.03% | 0.277 | 0.428 |
| Lobule 8: Pyramis | ↓ | 8.68% | 0.278 | 0.442 |
| Olfactory Bulbs | ↑ | 8.44% | 0.278 | 0.447 |
| Frontal Cortex: Area 3 | ↑ | 8.41% | 0.279 | 0.447 |
| Colliculus: Superior | ↓ | 8.26% | 0.281 | 0.450 |
| Secondary Visual Cortex: Mediolateral Area | ↑ | 7.96% | 0.283 | 0.463 |
| Paramedian Lobule: Lobule 7 | ↓ | 7.46% | 0.283 | 0.490 |
| Flocculus: Fl | ↓ | 7.24% | 0.285 | 0.496 |
| Secondary Visual Cortex: Mediomedial Area | ↑ | 7.03% | 0.294 | 0.496 |
| Mammilothalamic Tract | ↑ | 6.95% | 0.296 | 0.496 |
| Lobule 9: Uvula | ↓ | 6.89% | 0.312 | 0.496 |
| Simple Lobule White Matter | ↓ | 6.87% | 0.316 | 0.496 |
| Rostral Amygdalopiriform Area | ↑ | 6.83% | 0.316 | 0.496 |
| Secondary Auditory Cortex: Dorsal Area | ↑ | 6.57% | 0.318 | 0.498 |
| Anterior Lobule: Lobules 4 5 | ↓ | 6.55% | 0.335 | 0.498 |
| Lateral Olfactory Tract | ↓ | 6.44% | 0.339 | 0.498 |
| Paramedian Lobule | ↓ | 6.39% | 0.343 | 0.498 |
| Caudomedial Entorhinal Cortex | ↓ | 6.38% | 0.357 | 0.498 |
| Cerebellar Peduncle: Superior | ↑ | 6.20% | 0.357 | 0.505 |
| Crus 2: Ansiform Lobule: Lobule 7 | ↓ | 6.07% | 0.369 | 0.509 |
| Anterior Commissure: Pars Anterior | ↑ | 5.49% | 0.373 | 0.542 |
| Fasciculus Retroflexus | ↑ | 5.49% | 0.377 | 0.542 |
| Anterior Lobule White Matter | ↓ | 5.38% | 0.389 | 0.545 |
| Lobule 8 White Matter | ↓ | 5.17% | 0.394 | 0.556 |
| Lateral Parietal Association Cortex | ↑ | 4.92% | 0.399 | 0.564 |
| Dentate Gyrus Of Hippocampus | ↑ | 4.89% | 0.402 | 0.564 |
| Primary Somatosensory Cortex: Upper Lip Region | ↓ | 4.81% | 0.426 | 0.564 |
| Fundus Of Striatum | ↑ | 4.79% | 0.435 | 0.564 |
| Flocculus White Matter | ↓ | 4.50% | 0.435 | 0.580 |
| Primary Somatosensory Cortex | ↑ | 4.48% | 0.438 | 0.580 |
| Cingulate Cortex: Area 24A | ↑ | 4.35% | 0.450 | 0.580 |
| Corpus Callosum | ↑ | 4.34% | 0.453 | 0.580 |
| Primary Somatosensory Cortex: Jaw Region | ↓ | 4.18% | 0.453 | 0.584 |
| Dorsolateral Entorhinal Cortex | ↑ | 4.17% | 0.459 | 0.584 |
| Cingulate Cortex: Area 29B | ↓ | 3.93% | 0.461 | 0.597 |
| Primary Somatosensory Cortex: Trunk Region | ↑ | 3.88% | 0.465 | 0.597 |
| Lobules 6 7 White Matter | ↓ | 3.85% | 0.466 | 0.597 |
| Medial Parietal Association Cortex | ↑ | 3.54% | 0.467 | 0.615 |
| Lobule 6: Declive | ↓ | 3.54% | 0.471 | 0.615 |
| Primary Somatosensory Cortex: Dysgranular Zone | ↓ | 3.49% | 0.487 | 0.615 |
| Medial Lemniscus Medial Longitudinal Fasciculus | ↓ | 3.19% | 0.488 | 0.640 |
| Cingulate Cortex: Area 24B | ↑ | 3.03% | 0.498 | 0.644 |
| Secondary Visual Cortex: Lateral Area | ↑ | 2.98% | 0.505 | 0.644 |
| Lobule 1 2 White Matter | ↑ | 2.93% | 0.520 | 0.644 |
| Fornix | ↑ | 2.90% | 0.523 | 0.644 |
| Perirhinal Cortex | ↑ | 2.88% | 0.524 | 0.644 |
| Parietal Cortex: Posterior Area: Rostral Part | ↑ | 2.48% | 0.530 | 0.677 |
| Intermediate Nucleus Of The Endopiriform Claustrum | ↑ | 2.46% | 0.536 | 0.677 |
| Primary Visual Cortex: Binocular Area | ↑ | 2.45% | 0.556 | 0.677 |
| Secondary Somatosensory Cortex | ↓ | 2.39% | 0.559 | 0.678 |
| Medial Entorhinal Cortex | ↑ | 2.33% | 0.564 | 0.680 |
| Olfactory Tubercle | ↓ | 2.27% | 0.568 | 0.681 |
| Primary Somatosensory Cortex: Forelimb Region | ↓ | 2.00% | 0.585 | 0.711 |
| Thalamus | ↓ | 1.80% | 0.604 | 0.732 |
| Mammillary Bodies | ↓ | 1.62% | 0.608 | 0.746 |
| Primary Motor Cortex | ↑ | 1.56% | 0.610 | 0.746 |
| Cingulum | ↑ | 1.53% | 0.618 | 0.746 |
| Lobules 4 5: Culmen: Ventral And Dorsal | ↓ | 1.50% | 0.649 | 0.746 |
| Ventral Tegmental Decussation | ↓ | 1.48% | 0.653 | 0.746 |
| Cortex Amygdala Transition Zones | ↓ | 1.45% | 0.670 | 0.746 |
| Cerebral Aqueduct | ↑ | 1.44% | 0.701 | 0.746 |
| Crus 2 White Matter | ↑ | 1.37% | 0.729 | 0.748 |
| Internal Capsule | ↑ | 1.34% | 0.731 | 0.748 |
| Inferior Olivary Complex | ↓ | 1.31% | 0.734 | 0.748 |
| Lobules 4 5 White Matter | ↑ | 1.21% | 0.742 | 0.755 |
| Primary Auditory Cortex | ↑ | 1.20% | 0.744 | 0.755 |
| Lobule 3: Central Lobule: Dorsal | ↓ | 1.04% | 0.752 | 0.767 |
| Primary Somatosensory Cortex: Shoulder Region | ↓ | 1.04% | 0.753 | 0.767 |
| Cingulate Cortex: Area 29C | ↑ | 1.01% | 0.765 | 0.767 |
| Colliculus: Inferior | ↓ | 1.01% | 0.789 | 0.767 |
| Claustrum: Dorsal Part | ↑ | 0.87% | 0.806 | 0.787 |
| Trunk Of Lobules 6 8 White Matter | ↓ | 0.74% | 0.817 | 0.807 |
| Lobule 3 White Matter | ↑ | 0.69% | 0.818 | 0.813 |
| Primary Visual Cortex: Monocular Area | ↑ | 0.64% | 0.825 | 0.819 |
| Midbrain | ↑ | 0.54% | 0.832 | 0.835 |
| Ectorhinal Cortex | ↑ | 0.49% | 0.833 | 0.838 |
| Trunk Of Lobules 1 3 White Matter | ↑ | 0.49% | 0.841 | 0.838 |
| Stratum Granulosum Of Hippocampus | ↑ | 0.34% | 0.848 | 0.875 |
| Secondary Auditory Cortex: Ventral Area | ↓ | 0.24% | 0.850 | 0.900 |
| Primary Somatosensory Cortex: Barrel Field | ↓ | 0.20% | 0.851 | 0.912 |
| Trunk Of Simple And Crus 1 White Matter | ↑ | 0.18% | 0.865 | 0.913 |
| Primary Somatosensory Cortex: Hindlimb Region | ↓ | 0.15% | 0.867 | 0.913 |
| Stria Terminalis | ↓ | 0.15% | 0.874 | 0.913 |
| Temporal Association Area | ↓ | 0.14% | 0.890 | 0.914 |
| Fimbria | ↑ | 0.10% | 0.911 | 0.929 |
| Pre Para Subiculum | ↑ | 0.06% | 0.917 | 0.943 |
| Third Ventricle | ↓ | 0.03% | 0.917 | 0.964 |
| Cingulate Cortex: Area 30 | ↑ | 0.01% | 0.917 | 0.980 |
| Periaqueductal Grey | ↑ | 0.00% | 0.950 | 0.993 |
| Primary Visual Cortex | ↑ | 0.00% | 0.953 | 0.996 |
| Lobules 1 2: Lingula And Central Lobule: Ventral | ↓ | 0.00% | 0.994 | 0.996 |

**Supplementary Table 3: Amygdala's direct structural covariance neighboring nodes and edge weight (labeled as in Figure 5K and 5L).**

| **Control only** | **Edge Weight** | **Label (blue)** |
| --- | --- | --- |
| Ectorhinal Cortex | 0.772 | 1 |
| Temporal Association Area | 0.696 | 2 |
| Perirhinal Cortex | 0.690 | 3 |
| Secondary Visual Cortex: Lateral Area | 0.627 | 4 |
| Secondary Auditory Cortex: Ventral Area | 0.604 | 5 |
| Cingulate Cortex: Area 29C | 0.600 | 6 |
| Claustrum | 0.590 | 7 |
| Claustrum: Dorsal Part | 0.553 | 8 |
| Posterior Commissure | 0.504 | 9 |
| Medial Lemniscus/Medial Longitudinal Fasciculus | 0.447 | 10 |
| **Stress Only** | **Edge Weight** | **Label (red)** |
| Lobules 4-5 White Matter | 0.827 | 1 |
| Fasciculus Retroflexus | 0.809 | 2 |
| Posteromedial Cortical Amygdaloid Area | 0.777 | 3 |
| Ventral Intermediate Entorhinal Cortex | 0.748 | 4 |
| Anterior Commissure: Pars Anterior | 0.700 | 5 |
| Intermediate Nucleus Of The Endopiriform Claustrum | 0.695 | 6 |
| Lobules 6-7 White Matter | 0.694 | 7 |
| Lobule 1-2 White Matter | 0.664 | 8 |
| Anterior Commissure: Pars Posterior | 0.629 | 9 |
| Cortex-Amygdala Transition Zones | 0.627 | 10 |
| Basal Forebrain | 0.626 | 11 |
| Pre-Para Subiculum | 0.603 | 12 |
| Medial Entorhinal Cortex | 0.561 | 13 |
| Dentate Gyrus Of Hippocampus | 0.510 | 14 |
| Ventral Tenia Tecta | 0.498 | 15 |
| Olfactory Tubercle | 0.491 | 16 |
| Hypothalamus | 0.484 | 17 |
| Trunk Of Lobules 1-3 White Matter | 0.476 | 18 |
| Hippocampus | 0.472 | 19 |
| Bed Nucleus Of Stria Terminalis | 0.471 | 20 |
| Primary Somatosensory Cortex | 0.469 | 21 |
| Primary Visual Cortex: Monocular Area | 0.455 | 22 |
| Lobules 4-5: Culmen (Ventral And Dorsal) | 0.427 | 23 |
| Ventral Tegmental Decussation | 0.405 | 24 |
| Crus 1 White Matter | 0.395 | 25 |
| Cingulate Cortex: Area 24A | 0.362 | 26 |
| Frontal Cortex: Area 3 | 0.358 | 27 |
| Lobule 6: Declive | 0.341 | 28 |
| Trunk Of Simple And Crus 1 White Matter | 0.327 | 29 |
| Secondary Visual Cortex: Mediomedial Area | 0.320 | 30 |
| Frontal Association Cortex | 0.315 | 31 |
| Olfactory Bulbs | 0.311 | 32 |
| Mammilothalamic Tract | 0.307 | 33 |
| **Both Control and Stress**  **(edge weights from Stress)** | **Edge Weight** | **Label (purple)** |
| Ventral Nucleus Of The Endopiriform Claustrum | 0.870 | 1 |
| Amygdalopiriform Transition Area | 0.861 | 2 |
| Piriform Cortex | 0.845 | 3 |
| Dorsal Nucleus Of The Endopiriform | 0.767 | 4 |
| Posterolateral Cortical Amygdaloid Area | 0.764 | 5 |
| Globus Pallidus | 0.753 | 6 |
| Rostral Amygdalopiriform Area | 0.718 | 7 |
| Dorsal Intermediate Entorhinal Cortex | 0.716 | 8 |
| Insular Region: Not Subdivided | 0.632 | 9 |
| Fundus Of Striatum | 0.613 | 10 |
| Dorsolateral Orbital Cortex | 0.536 | 11 |
| Nucleus Accumbens | 0.526 | 12 |
| Subependymale Zone / Rhinocele | 0.494 | 13 |
| Medial Orbital Cortex | 0.490 | 14 |
| Lateral Orbital Cortex | 0.456 | 15 |
| Cingulate Cortex: Area 25 | 0.456 | 16 |
| Primary Visual Cortex: Binocular Area | 0.434 | 17 |
| Secondary Somatosensory Cortex | 0.411 | 18 |
| Dorsolateral Entorhinal Cortex | 0.407 | 19 |
| Claustrum: Ventral Part | 0.365 | 20 |
| Striatum | 0.350 | 21 |
| Cingulate Cortex: Area 32 | 0.335 | 22 |
| Dorsal Tenia Tecta | 0.318 | 23 |

**Supplementary Table 4:** Mouse brain regions and corresponding structural covariance degree (i.e., number of connections to other nodes) at 22% density, listed by degree rank in the Control (left) and Stress (right) group.

| **Control** | | |  | **Stress** | | |
| --- | --- | --- | --- | --- | --- | --- |
| **Degree rank** | **Brain Region** | **Degree** |  | **Degree rank** | **Brain Region** | **Degree** |
| 1 | Crus 1 White Matter | 58 |  | 1 | Lobules 4-5 White Matter | 62 |
| 2 | Crus 1: Ansiform Lobule (Lobule 6) | 56 |  | **2** | **Amygdala** | **56** |
| 3 | Simple Lobule White Matter | 56 |  | 3 | Lobules 6-7 White Matter | 54 |
| 4 | Olfactory Tubercle | 55 |  | 4 | Posterolateral Cortical Amygdaloid Area | 54 |
| 5 | Lobule 7: Tuber (Or Folium) | 55 |  | 5 | Rostral Amygdalopiriform Area | 54 |
| 6 | Lobule 3 White Matter | 55 |  | 6 | Amygdalopiriform Transition Area | 53 |
| 7 | Trunk Of Lobules 6-8 White Matter | 55 |  | 7 | Insular Region: Not Subdivided | 53 |
| 8 | Colliculus: Superior | 54 |  | 8 | Globus Pallidus | 52 |
| 9 | Lobule 9: Uvula | 54 |  | 9 | Medial Orbital Cortex | 52 |
| 10 | Anterior Lobule (Lobules 4-5) | 54 |  | 10 | Fasciculus Retroflexus | 50 |
| 11 | Lobule 1-2 White Matter | 54 |  | 11 | Piriform Cortex | 50 |
| 12 | Anterior Lobule White Matter | 54 |  | 12 | Ventral Nucleus Of The Endopiriform Claustrum | 50 |
| 13 | Crus 2 White Matter | 54 |  | 13 | Basal Forebrain | 49 |
| 14 | Dentate Nucleus | 54 |  | 14 | Ventral Tenia Tecta | 49 |
| 15 | Basal Forebrain | 53 |  | 15 | Dentate Gyrus Of Hippocampus | 48 |
| 16 | Cerebellar Peduncle: Middle | 53 |  | 16 | Trunk Of Simple And Crus 1 White Matter | 48 |
| 17 | Simple Lobule (Lobule 6) | 53 |  | 17 | Dorsolateral Orbital Cortex | 48 |
| 18 | Trunk Of Arbor Vita | 53 |  | 18 | Intermediate Nucleus Of The Endopiriform Claustrum | 48 |
| 19 | Trunk Of Crus 2 And Paramedian White Matter | 53 |  | 19 | Posteromedial Cortical Amygdaloid Area | 48 |
| 20 | Trunk Of Lobules 1-3 White Matter | 52 |  | 20 | Bed Nucleus Of Stria Terminalis | 47 |
| 21 | Lobule 8 White Matter | 52 |  | 21 | Fundus Of Striatum | 47 |
| 22 | Copula White Matter | 52 |  | 22 | Hypothalamus | 47 |
| 23 | Flocculus White Matter | 52 |  | 23 | Subependymale Zone / Rhinocele | 47 |
| 24 | Ventral Tenia Tecta | 52 |  | 24 | Lobule 1-2 White Matter | 47 |
| 25 | Paramedian Lobule (Lobule 7) | 51 |  | 25 | Crus 1 White Matter | 47 |
| 26 | Copula: Pyramis (Lobule 8) | 51 |  | 26 | Cingulate Cortex: Area 32 | 47 |
| 27 | Lobules 6-7 White Matter | 51 |  | 27 | Olfactory Tubercle | 46 |
| 28 | Trunk Of Simple And Crus 1 White Matter | 51 |  | 28 | Trunk Of Lobules 1-3 White Matter | 46 |
| 29 | Nucleus Interpositus | 51 |  | 29 | Cingulate Cortex: Area 25 | 46 |
| 30 | Optic Tract | 50 |  | 30 | Dorsal Nucleus Of The Endopiriform | 46 |
| 31 | Pontine Nucleus | 50 |  | 31 | Ventral Tegmental Decussation | 45 |
| 32 | Lobule 3: Central Lobule (Dorsal) | 50 |  | 32 | Primary Visual Cortex: Binocular Area | 44 |
| 33 | Lobule 6: Declive | 50 |  | 33 | Corpus Callosum | 43 |
| 34 | Lobules 4-5 White Matter | 50 |  | 34 | Lobule 3 White Matter | 43 |
| 35 | Lobule 8: Pyramis | 49 |  | 35 | Dorsal Tenia Tecta | 43 |
| 36 | Lobule 10: Nodulus | 49 |  | 36 | Primary Visual Cortex: Monocular Area | 43 |
| 37 | Flocculus (Fl) | 49 |  | 37 | Ventral Orbital Cortex | 43 |
| 38 | Paraflocculus White Matter | 49 |  | 38 | Anterior Commissure: Pars Anterior | 42 |
| 39 | Fastigial Nucleus | 49 |  | 39 | Mammilothalamic Tract | 42 |
| 40 | Mammilothalamic Tract | 48 |  | 40 | Nucleus Accumbens | 42 |
| 41 | Stratum Granulosum Of Hippocampus | 48 |  | 41 | Claustrum: Ventral Part | 42 |
| 42 | Lobule 9 White Matter | 48 |  | 42 | Ventral Intermediate Entorhinal Cortex | 42 |
| 43 | Lobules 4-5: Culmen (Ventral And Dorsal) | 47 |  | 43 | Anterior Commissure: Pars Posterior | 41 |
| 44 | Lateral Olfactory Tract | 43 |  | 44 | Frontal Association Cortex | 41 |
| 45 | Crus 2: Ansiform Lobule (Lobule 7) | 43 |  | 45 | Lateral Orbital Cortex | 41 |
| 46 | Lobules 1-2: Lingula And Central Lobule (Ventral) | 40 |  | 46 | Cingulate Cortex: Area 24A | 40 |
| 47 | Cingulate Cortex: Area 32 | 40 |  | 47 | Cortex-Amygdala Transition Zones | 40 |
| 48 | Corpus Callosum | 39 |  | 48 | Primary Somatosensory Cortex | 40 |
| 49 | Fasciculus Retroflexus | 39 |  | 49 | Secondary Visual Cortex: Mediomedial Area | 40 |
| 50 | Cingulate Cortex: Area 29B | 39 |  | 50 | Cingulum | 39 |
| 51 | Olfactory Bulbs | 38 |  | 51 | Frontal Cortex: Area 3 | 39 |
| 52 | Anterior Commissure: Pars Anterior | 37 |  | 52 | Pre-Para Subiculum | 37 |
| 53 | Cingulate Cortex: Area 29A | 37 |  | 53 | Striatum | 37 |
| 54 | Frontal Association Cortex | 37 |  | 54 | Lobule 3: Central Lobule (Dorsal) | 37 |
| 55 | Periaqueductal Grey | 36 |  | 55 | Cingulate Cortex: Area 29A | 37 |
| 56 | Mammillary Bodies | 35 |  | 56 | Claustrum: Dorsal Part | 37 |
| 57 | Subependymale Zone / Rhinocele | 35 |  | 57 | Cerebral Peduncle | 36 |
| 58 | Paramedian Lobule | 35 |  | 58 | Internal Capsule | 35 |
| 59 | Posterolateral Cortical Amygdaloid Area | 34 |  | 59 | Olfactory Bulbs | 35 |
| **60** | **Amygdala** | **33** |  | 60 | Claustrum | 35 |
| 61 | Stria Medullaris | 33 |  | 61 | Primary Somatosensory Cortex: Trunk Region | 35 |
| 62 | Dorsal Nucleus Of The Endopiriform | 33 |  | 62 | Lobule 9 White Matter | 34 |
| 63 | Perirhinal Cortex | 33 |  | 63 | Paramedian Lobule | 34 |
| 64 | Rostral Amygdalopiriform Area | 33 |  | 64 | Medial Entorhinal Cortex | 34 |
| 65 | Cuneate Nucleus | 32 |  | 65 | Primary Somatosensory Cortex: Barrel Field | 34 |
| 66 | Medial Septum | 32 |  | 66 | Secondary Visual Cortex: Mediolateral Area | 34 |
| 67 | Cingulate Cortex: Area 25 | 32 |  | 67 | Hippocampus | 33 |
| 68 | Secondary Auditory Cortex: Dorsal Area | 32 |  | 68 | Lateral Septum | 33 |
| 69 | Cortex-Amygdala Transition Zones | 32 |  | 69 | Copula White Matter | 33 |
| 70 | Medial Orbital Cortex | 32 |  | 70 | Midbrain | 32 |
| 71 | Primary Visual Cortex: Binocular Area | 32 |  | 71 | Trunk Of Arbor Vita | 32 |
| 72 | Fundus Of Striatum | 31 |  | 72 | Trunk Of Lobules 6-8 White Matter | 32 |
| 73 | Dorsal Intermediate Entorhinal Cortex | 31 |  | 73 | Cingulate Cortex: Area 29B | 32 |
| 74 | Ectorhinal Cortex | 31 |  | 74 | Secondary Auditory Cortex: Dorsal Area | 32 |
| 75 | Lateral Orbital Cortex | 31 |  | 75 | Parietal Cortex: Posterior Area: Rostral Part | 32 |
| 76 | Secondary Visual Cortex: Lateral Area | 31 |  | 76 | Cerebellar Peduncle: Middle | 31 |
| 77 | Anterior Commissure: Pars Posterior | 30 |  | 77 | Habenular Commissure | 31 |
| 78 | Internal Capsule | 30 |  | 78 | Pons | 31 |
| 79 | Striatum | 30 |  | 79 | Superior Olivary Complex | 31 |
| 80 | Cingulate Cortex: Area 24B | 30 |  | 80 | Lobule 9: Uvula | 31 |
| 81 | Cingulum | 30 |  | 81 | Paramedian Lobule (Lobule 7) | 31 |
| 82 | Dorsolateral Entorhinal Cortex | 30 |  | 82 | Copula: Pyramis (Lobule 8) | 31 |
| 83 | Claustrum: Ventral Part | 30 |  | 83 | Paraflocculus White Matter | 31 |
| 84 | Nucleus Accumbens | 29 |  | 84 | Dorsal Intermediate Entorhinal Cortex | 31 |
| 85 | Secondary Auditory Cortex: Ventral Area | 29 |  | 85 | Dorsolateral Entorhinal Cortex | 31 |
| 86 | Dorsolateral Orbital Cortex | 29 |  | 86 | Lateral Olfactory Tract | 30 |
| 87 | Medial Entorhinal Cortex | 29 |  | 87 | Stria Terminalis | 30 |
| 88 | Medial Parietal Association Cortex | 29 |  | 88 | Dentate Nucleus | 30 |
| 89 | Piriform Cortex | 29 |  | 89 | Cingulate Cortex: Area 30 | 30 |
| 90 | Temporal Association Area | 29 |  | 90 | Secondary Motor Cortex | 30 |
| 91 | Claustrum | 28 |  | 91 | Perirhinal Cortex | 30 |
| 92 | Dorsal Tenia Tecta | 28 |  | 92 | Primary Somatosensory Cortex: Shoulder Region | 30 |
| 93 | Lateral Parietal Association Cortex | 28 |  | 93 | Cerebellar Peduncle: Inferior | 29 |
| 94 | Secondary Motor Cortex | 28 |  | 94 | Stratum Granulosum Of Hippocampus | 29 |
| 95 | Primary Somatosensory Cortex: Jaw Region | 28 |  | 95 | Lobules 4-5: Culmen (Ventral And Dorsal) | 29 |
| 96 | Primary Visual Cortex: Monocular Area | 28 |  | 96 | Crus 2 White Matter | 29 |
| 97 | Globus Pallidus | 27 |  | 97 | Ectorhinal Cortex | 29 |
| 98 | Stria Terminalis | 27 |  | 98 | Lateral Parietal Association Cortex | 29 |
| 99 | Lobule 10 White Matter | 27 |  | 99 | Secondary Visual Cortex: Lateral Area | 29 |
| 100 | Insular Region: Not Subdivided | 27 |  | 100 | Medial Lemniscus/Medial Longitudinal Fasciculus | 28 |
| 101 | Posteromedial Cortical Amygdaloid Area | 27 |  | 101 | Optic Tract | 28 |
| 102 | Primary Somatosensory Cortex | 27 |  | 102 | Pontine Nucleus | 28 |
| 103 | Cingulate Cortex: Area 29C | 26 |  | 103 | Crus 1: Ansiform Lobule (Lobule 6) | 28 |
| 104 | Primary Motor Cortex | 26 |  | 104 | Secondary Somatosensory Cortex | 28 |
| 105 | Primary Somatosensory Cortex: Hindlimb Region | 26 |  | 105 | Lobule 10: Nodulus | 27 |
| 106 | Secondary Visual Cortex: Mediolateral Area | 26 |  | 106 | Simple Lobule (Lobule 6) | 27 |
| 107 | Ventral Nucleus Of The Endopiriform Claustrum | 26 |  | 107 | Flocculus (Fl) | 27 |
| 108 | Ventral Intermediate Entorhinal Cortex | 26 |  | 108 | Lobule 10 White Matter | 27 |
| 109 | Bed Nucleus Of Stria Terminalis | 25 |  | 109 | Cerebellar Peduncle: Superior | 26 |
| 110 | Cerebellar Peduncle: Inferior | 25 |  | 110 | Colliculus: Superior | 26 |
| 111 | Medial Lemniscus/Medial Longitudinal Fasciculus | 25 |  | 111 | Fornix | 26 |
| 112 | Medulla | 25 |  | 112 | Inferior Olivary Complex | 26 |
| 113 | Amygdalopiriform Transition Area | 25 |  | 113 | Interpedunclar Nucleus | 26 |
| 114 | Intermediate Nucleus Of The Endopiriform Claustrum | 25 |  | 114 | Lobule 7: Tuber (Or Folium) | 26 |
| 115 | Secondary Somatosensory Cortex | 25 |  | 115 | Anterior Lobule White Matter | 26 |
| 116 | Ventral Orbital Cortex | 25 |  | 116 | Trunk Of Crus 2 And Paramedian White Matter | 26 |
| 117 | Lateral Septum | 24 |  | 117 | Primary Auditory Cortex | 26 |
| 118 | Cingulate Cortex: Area 24A | 24 |  | 118 | Corticospinal Tract/Pyramids | 25 |
| 119 | Primary Somatosensory Cortex: Barrel Field | 24 |  | 119 | Facial Nerve (Cranial Nerve 7) | 25 |
| 120 | Primary Somatosensory Cortex: Dysgranular Zone | 24 |  | 120 | Medial Septum | 25 |
| 121 | Primary Somatosensory Cortex: Forelimb Region | 24 |  | 121 | Posterior Commissure | 25 |
| 122 | Dentate Gyrus Of Hippocampus | 23 |  | 122 | Stria Medullaris | 25 |
| 123 | Claustrum: Dorsal Part | 23 |  | 123 | Lobule 8 White Matter | 25 |
| 124 | Corticospinal Tract/Pyramids | 22 |  | 124 | Secondary Auditory Cortex: Ventral Area | 25 |
| 125 | Inferior Olivary Complex | 22 |  | 125 | Primary Somatosensory Cortex: Dysgranular Zone | 25 |
| 126 | Parietal Cortex: Posterior Area: Rostral Part | 22 |  | 126 | Temporal Association Area | 25 |
| 127 | Primary Somatosensory Cortex: Shoulder Region | 22 |  | 127 | Mammillary Bodies | 24 |
| 128 | Primary Somatosensory Cortex: Trunk Region | 22 |  | 128 | Periaqueductal Grey | 24 |
| 129 | Secondary Visual Cortex: Mediomedial Area | 22 |  | 129 | Lobule 6: Declive | 24 |
| 130 | Facial Nerve (Cranial Nerve 7) | 21 |  | 130 | Lobule 8: Pyramis | 24 |
| 131 | Hypothalamus | 21 |  | 131 | Flocculus White Matter | 24 |
| 132 | Ventral Tegmental Decussation | 21 |  | 132 | Primary Visual Cortex | 24 |
| 133 | Cingulate Cortex: Area 24B' | 21 |  | 133 | Colliculus: Inferior | 23 |
| 134 | Primary Auditory Cortex | 21 |  | 134 | Cuneate Nucleus | 23 |
| 135 | Primary Visual Cortex | 21 |  | 135 | Fimbria | 23 |
| 136 | Fimbria | 20 |  | 136 | Thalamus | 23 |
| 137 | Posterior Commissure | 20 |  | 137 | Anterior Lobule (Lobules 4-5) | 23 |
| 138 | Superior Olivary Complex | 20 |  | 138 | Cingulate Cortex: Area 24B | 23 |
| 139 | Cingulate Cortex: Area 24A' | 20 |  | 139 | Primary Motor Cortex | 23 |
| 140 | Cingulate Cortex: Area 30 | 20 |  | 140 | Medial Parietal Association Cortex | 23 |
| 141 | Caudomedial Entorhinal Cortex | 20 |  | 141 | Primary Somatosensory Cortex: Forelimb Region | 23 |
| 142 | Fornix | 19 |  | 142 | Primary Somatosensory Cortex: Upper Lip Region | 23 |
| 143 | Habenular Commissure | 19 |  | 143 | Medulla | 22 |
| 144 | Cerebral Peduncle | 18 |  | 144 | Lobules 1-2: Lingula And Central Lobule (Ventral) | 22 |
| 145 | Pons | 18 |  | 145 | Crus 2: Ansiform Lobule (Lobule 7) | 22 |
| 146 | Pre-Para Subiculum | 18 |  | 146 | Paraflocculus (Pfl) | 22 |
| 147 | Paraflocculus (Pfl) | 18 |  | 147 | Nucleus Interpositus | 22 |
| 148 | Frontal Cortex: Area 3 | 18 |  | 148 | Caudomedial Entorhinal Cortex | 22 |
| 149 | Cerebellar Peduncle: Superior | 17 |  | 149 | Primary Somatosensory Cortex: Hindlimb Region | 21 |
| 150 | Midbrain | 17 |  | 150 | Fastigial Nucleus | 20 |
| 151 | Hippocampus | 13 |  | 151 | Cingulate Cortex: Area 29C | 20 |
| 152 | Interpedunclar Nucleus | 13 |  | 152 | Cingulate Cortex: Area 24A' | 19 |
| 153 | Thalamus | 13 |  | 153 | Cingulate Cortex: Area 24B' | 18 |
| 154 | Colliculus: Inferior | 12 |  | 154 | Simple Lobule White Matter | 15 |
| 155 | Primary Somatosensory Cortex: Upper Lip Region | 11 |  | 155 | Primary Somatosensory Cortex: Jaw Region | 15 |

Note: The amygdala is highlighted in bold. The order of the brain regions (degree rank) correspond to the order depicted in the circular network plots illustrating the mouse structural covariance network for control (Figure 5E) and stress group (Figure 5F).

| **Supplementary Table 5**: Demographic characteristics and depression/anxiety symptoms in the human sample (Duke Neurogenetics Study) presented as mean ± standard deviation. |
| --- |

| **Demographics** | **Low CTQ**  **(<27, n=237)** | **High CTQ**  **(>37, n=299)** | **t / Chi Square** | **p value** |
| --- | --- | --- | --- | --- |
| **Age** | 19.81 ± 1.29 | 19.53 ± 1.26 | 2.51 | 0.01 Ϯ |
| **Female** | 60.34% | 59.20% | 0.0714 | 0.789 |
| **MASQ GDA** | 16.24 ± 5.06 | 20.39 ± 7.43 | -7.51 | <0.001 |
| **MASQ GDD** | 18.09 ± 6.39 | 25.58 ± 10.48 | -10.19 | <0.001 |
| **MDD lifetime diagnosis** | 0.80% | 28.80% | 74.7868 | <0.0001 |

^Ϯ^ Please note that due to the limited age range (18-22) and large sample size, the high CTQ group is slightly, but significantly younger than the low CTQ group (19 years, 6 months vs. 9 months). However, we do not consider this difference biologically meaningful and do not control for it in our analyses.

**Supplementary Table 6:** Whole-brain between-group (control vs. stress) volumetric comparison results in the human sample, listed by significance (from smallest to largest uncorrected p value). Regional volumes were normalized by whole-brain volume for each individual prior to analysis.

| **Brain Region** | **Group Effects** | | | |
| --- | --- | --- | --- | --- |
|  | **Direction of Change** | **t** | **p** | **q** |
| **Occipital Fusiform Gyrus** | **↑** | **4.260** | **<0.001** | **0.001** |
| Parahippocampal Gyrus (anterior division) | ↑ | 3.165 | 0.002 | 0.090 |
| Frontal Orbital Cortex | ↑ | 2.852 | 0.005 | 0.244 |
| Temporal Fusiform Cortex (anterior division) | ↑ | 2.589 | 0.010 | 0.524 |
| Superior Frontal Gyrus | ↓ | -2.530 | 0.012 | 0.608 |
| Cuneal Cortex | ↑ | 2.504 | 0.013 | 0.642 |
| Temporal Occipital Fusiform Cortex | ↑ | 2.471 | 0.014 | 0.690 |
| Parahippocampal Gyrus (posterior division) | ↑ | 2.143 | 0.033 | 1 |
| Lingual Gyrus | ↑ | 1.917 | 0.056 | 1 |
| Thalamus | ↑ | 1.899 | 0.058 | 1 |
| Intracalcarine Cortex | ↑ | 1.760 | 0.079 | 1 |
| Supracalcarine Cortex | ↑ | 1.544 | 0.123 | 1 |
| Parietal Operculum Cortex | ↓ | -1.502 | 0.134 | 1 |
| Inferior Frontal Gyrus (pars opercularis) | ↓ | -1.447 | 0.149 | 1 |
| Juxtapositional Lobule Cortex (formerly Supplementary Motor Cortex) | ↓ | -1.432 | 0.153 | 1 |
| Temporal Pole | ↑ | 1.313 | 0.190 | 1 |
| Inferior Temporal Gyrus (temporooccipital part) | ↑ | 1.295 | 0.196 | 1 |
| Lateral Occipital Cortex (inferior division) | ↑ | 1.182 | 0.238 | 1 |
| Temporal Fusiform Cortex (posterior division) | ↑ | 1.137 | 0.256 | 1 |
| Middle Frontal Gyrus | ↓ | -1.118 | 0.264 | 1 |
| Accumbens | ↓ | -1.059 | 0.290 | 1 |
| Putamen | ↑ | 1.042 | 0.298 | 1 |
| Occipital Pole | ↑ | 1.033 | 0.302 | 1 |
| Paracingulate Gyrus | ↓ | -1.013 | 0.312 | 1 |
| Supramarginal Gyrus (anterior division) | ↓ | -0.956 | 0.340 | 1 |
| Precuneous Cortex | ↑ | 0.955 | 0.340 | 1 |
| Hippocampus | ↓ | -0.929 | 0.353 | 1 |
| Pallidum | ↑ | 0.798 | 0.425 | 1 |
| Postcentral Gyrus | ↓ | -0.729 | 0.466 | 1 |
| Frontal Operculum Cortex | ↓ | -0.703 | 0.482 | 1 |
| Subcallosal Cortex | ↓ | -0.692 | 0.489 | 1 |
| Supramarginal Gyrus (posterior division) | ↓ | -0.541 | 0.589 | 1 |
| Lateral Occipital Cortex (superior division) | ↓ | -0.517 | 0.606 | 1 |
| Frontal Medial Cortex | ↓ | -0.516 | 0.606 | 1 |
| Cingulate Gyrus (posterior division) | ↓ | -0.507 | 0.612 | 1 |
| Brain Stem | ↑ | 0.496 | 0.620 | 1 |
| Middle Temporal Gyrus (anterior division) | ↑ | 0.489 | 0.625 | 1 |
| Inferior Temporal Gyrus (posterior division) | ↓ | -0.478 | 0.633 | 1 |
| Planum Polare | ↓ | -0.471 | 0.638 | 1 |
| Precentral Gyrus | ↓ | -0.435 | 0.664 | 1 |
| Superior Parietal Lobule | ↓ | -0.397 | 0.692 | 1 |
| Inferior Frontal Gyrus (pars triangularis) | ↑ | 0.394 | 0.694 | 1 |
| Middle Temporal Gyrus (posterior division) | ↓ | -0.353 | 0.724 | 1 |
| Caudate | ↑ | 0.345 | 0.730 | 1 |
| Heschl's Gyrus (includes H1 and H2) | ↑ | 0.322 | 0.747 | 1 |
| Middle Temporal Gyrus (temporooccipital part) | ↑ | 0.314 | 0.754 | 1 |
| Cingulate Gyrus (anterior division) | ↑ | 0.295 | 0.768 | 1 |
| Superior Temporal Gyrus (anterior division) | ↓ | -0.177 | 0.860 | 1 |
| Angular Gyrus | ↑ | 0.172 | 0.863 | 1 |
| Inferior Temporal Gyrus (anterior division) | ↓ | -0.129 | 0.897 | 1 |
| Central Opercular Cortex | ↓ | -0.128 | 0.898 | 1 |
| Superior Temporal Gyrus (posterior division) | ↓ | -0.127 | 0.899 | 1 |
| Planum Temporale | ↑ | 0.090 | 0.928 | 1 |
| Frontal Pole | ↑ | 0.075 | 0.940 | 1 |
| Insular Cortex | ↑ | 0.023 | 0.982 | 1 |
| Amygdala | ↓ | -0.013 | 0.990 | 1 |

Note: Regions highlighted in bold font show both an effect of Group at the q<0.05 significance level.

**Supplementary Table 7: Human brain regions and corresponding structural covariance degree (i.e., number of connections to other nodes) at 22% density, listed by degree rank in the Control (left) and Stress (right) group.**

| **Control** | | |  | **Stress** | | |
| --- | --- | --- | --- | --- | --- | --- |
| **Degree Rank** | **Brain Region** | **Degree** |  | **Degree Rank** | **Brain Region** | **Degree** |
| 1 | Superior Temporal Gyrus (anterior division) | 17 |  | 1 | Superior Temporal Gyrus (posterior division) | 18 |
| 2 | Superior Temporal Gyrus (posterior division) | 17 |  | 2 | Superior Temporal Gyrus (anterior division) | 17 |
| 3 | Middle Temporal Gyrus (anterior division) | 14 |  | 3 | Temporal Fusiform Cortex (posterior division) | 14 |
| 4 | Temporal Fusiform Cortex (posterior division) | 13 |  | 4 | Temporal Pole | 13 |
| 5 | Inferior Frontal Gyrus (pars triangularis) | 12 |  | 5 | Inferior Temporal Gyrus (anterior division) | 13 |
| 6 | Inferior Temporal Gyrus (anterior division) | 12 |  | 6 | Middle Temporal Gyrus (anterior division) | 13 |
| 7 | Temporal Pole | 12 |  | 7 | Parahippocampal Gyrus (posterior division) | 12 |
| 8 | Middle Temporal Gyrus temporooccipital part | 11 |  | 8 | Temporal Fusiform Cortex (anterior division) | 12 |
| 9 | Parahippocampal Gyrus (posterior division) | 11 |  | 9 | Middle Temporal Gyrus (posterior division) | 12 |
| 10 | Inferior Temporal Gyrus (posterior division) | 10 |  | 10 | Inferior Frontal Gyrus (pars triangularis) | 12 |
| 11 | Middle Temporal Gyrus (posterior division) | 10 |  | 11 | Parahippocampal Gyrus (anterior division) | 11 |
| 12 | Frontal Operculum Cortex | 9 |  | 12 | Inferior Temporal Gyrus (posterior division) | 11 |
| 13 | Inferior Temporal Gyrus temporooccipital part | 9 |  | 13 | Lingual Gyrus | 10 |
| 14 | Supramarginal Gyrus (anterior division) | 9 |  | 14 | Middle Temporal Gyrus temporooccipital part | 9 |
| 15 | Angular Gyrus | 8 |  | 15 | Cuneal Cortex | 9 |
| 16 | Parahippocampal Gyrus (anterior division) | 8 |  | **16** | **Amygdala** | **8** |
| 17 | Supramarginal Gyrus (posterior division) | 8 |  | 17 | Supramarginal Gyrus (anterior division) | 8 |
| 18 | Insular Cortex | 7 |  | 18 | Hippocampus | 7 |
| 19 | Temporal Fusiform Cortex (anterior division) | 7 |  | 19 | Central Opercular Cortex | 7 |
| 20 | Temporal Occipital Fusiform Cortex | 7 |  | 20 | Planum Temporale | 7 |
| 21 | Central Opercular Cortex | 6 |  | 21 | Insular Cortex | 6 |
| 22 | Cuneal Cortex | 6 |  | 22 | Angular Gyrus | 6 |
| 23 | Heschl s Gyrus (includes H1 and H2) | 6 |  | 23 | Supracalcarine Cortex | 6 |
| 24 | Lateral Occipital Cortex inferior division) | 6 |  | 24 | Precentral Gyrus | 6 |
| 25 | Lingual Gyrus | 6 |  | 25 | Accumbens | 5 |
| 26 | Occipital Pole | 6 |  | 26 | Pallidum | 5 |
| 27 | Parietal Operculum Cortex | 6 |  | 27 | Putamen | 5 |
| 28 | Planum Polare | 6 |  | 28 | Subcallosal Cortex | 5 |
| 29 | Planum Temporale | 6 |  | 29 | Supramarginal Gyrus (posterior division) | 5 |
| 30 | Supracalcarine Cortex | 6 |  | 30 | Heschl s Gyrus (includes H1 and H2) | 5 |
| 31 | Lateral Occipital Cortex (superior division) | 5 |  | 31 | Planum Polare | 5 |
| 32 | Occipital Fusiform Gyrus | 5 |  | 32 | Frontal Medial Cortex | 5 |
| 33 | Paracingulate Gyrus | 5 |  | 33 | Paracingulate Gyrus | 5 |
| 34 | Precentral Gyrus | 5 |  | 34 | Lateral Occipital Cortex inferior division) | 5 |
| 35 | Putamen | 5 |  | 35 | Occipital Pole | 5 |
| 36 | Superior Parietal Lobule | 5 |  | 36 | Postcentral Gyrus | 5 |
| 37 | Accumbens | 4 |  | 37 | Inferior Temporal Gyrus temporooccipital part | 5 |
| 38 | Caudate | 4 |  | 38 | Temporal Occipital Fusiform Cortex | 5 |
| 39 | Cingulate Gyrus (anterior division) | 4 |  | 39 | Frontal Operculum Cortex | 4 |
| 40 | Frontal Medial Cortex | 4 |  | 40 | Inferior Frontal Gyrus (pars opercularis) | 4 |
| 41 | Frontal Orbital Cortex | 4 |  | 41 | Cingulate Gyrus (anterior division) | 4 |
| 42 | Frontal Pole | 4 |  | 42 | Juxtapositional Lobule Cortex (formerly Supplementary Motor Cortex) | 4 |
| 43 | Hippocampus | 4 |  | 43 | Intracalcarine Cortex | 4 |
| 44 | Inferior Frontal Gyrus (pars opercularis) | 4 |  | 44 | Occipital Fusiform Gyrus | 4 |
| 45 | Intracalcarine Cortex | 4 |  | 45 | Frontal Pole | 4 |
| 46 | Pallidum | 4 |  | 46 | Lateral Occipital Cortex (superior division) | 3 |
| 47 | Subcallosal Cortex | 4 |  | 47 | Parietal Operculum Cortex | 3 |
| 48 | **Amygdala** | **3** |  | 48 | Precuneous Cortex | 3 |
| 49 | Cingulate Gyrus (posterior division) | 3 |  | 49 | Superior Frontal Gyrus | 3 |
| 50 | Juxtapositional Lobule Cortex (formerly Supplementary Motor Cortex) | 3 |  | 50 | Superior Parietal Lobule | 3 |
| 51 | Postcentral Gyrus | 3 |  | 51 | Caudate | 2 |
| 52 | Precuneous Cortex | 3 |  | 52 | Frontal Orbital Cortex | 2 |
| 53 | Superior Frontal Gyrus | 3 |  | 53 | Middle Frontal Gyrus | 2 |
| 54 | Thalamus | 3 |  | 54 | Brain Stem | 2 |
| 55 | Brain Stem | 2 |  | 55 | Cingulate Gyrus (posterior division) | 1 |
| 56 | Middle Frontal Gyrus | 2 |  | 56 | Thalamus | 1 |

Note: The amygdala is highlighted in bold. The order of the brain regions (degree rank) correspond to the order depicted in the circular network plots illustrating the human structural covariance network for control (Figure 5G) and early stress individuals (Figure 5H).

**References**

1. Soumier A, Sibille E. Opposing effects of acute versus chronic blockade of frontal cortex somatostatin-positive inhibitory neurons on behavioral emotionality in mice. *Neuropsychopharmacology* 2014; **39:** 2252-2262.

2. Bernstein D, Stein, JA, Newcomb, MD, Walker, E, Pogge, D, Ahluvalia, T, Stokes, J, Handelsman, L, Medrano, M, Desmond, D, and Zule, W. Development and validation of a brief screening version of the Childhood Trauma Questionnaire. *Child Abuse Negl* 2002; **27:** 169-190.

3. Watson D, Clark LA, Weber K, Assenheimer JS, Strauss ME, McCormick RA. Testing a tripartite model: II. Exploring the symptom structure of anxiety and depression in student, adult, and patient samples. *J Abnorm Psychol* 1995; **104:** 15-25.

4. Sheehan DV, Lecrubier Y, Sheehan KH, Amorim P, Janavs J, Weiller E *et al.* The Mini-International Neuropsychiatric Interview (M.I.N.I.): the development and validation of a structured diagnostic psychiatric interview for DSM-IV and ICD-10. *J Clin Psychiatry* 1998; **59 Suppl 20:** 22-33;quiz 34-57.

5. Nikolova YS, Bogdan R, Brigidi BD, Hariri AR. Ventral striatum reactivity to reward and recent life stress interact to predict positive affect. *Biol Psychiatry* 2012; **72:** 157-163.

6. Cahill LS, Laliberte CL, Ellegood J, Spring S, Gleave JA, Eede MC *et al.* Preparation of fixed mouse brains for MRI. *Neuroimage* 2012; **60:** 933-939.

7. Lerch JP, Sled JG, Henkelman RM. MRI phenotyping of genetically altered mice. *Methods Mol Biol* 2011; **711:** 349-361.

8. Dazai J, Spring S, Cahill LS, Henkelman RM. Multiple-mouse neuroanatomical magnetic resonance imaging. *J Vis Exp* 2011.

9. Nieman BJ, Bock NA, Bishop J, Sled JG, Josette Chen X, Mark Henkelman R. Fast spin-echo for multiple mouse magnetic resonance phenotyping. *Magn Reson Med* 2005; **54:** 532-537.

10. Collins DL, Neelin P, Peters TM, Evans AC. Automatic 3D intersubject registration of MR volumetric data in standardized Talairach space. *J Comput Assist Tomogr* 1994; **18:** 192-205.

11. Avants BB, Epstein CL, Grossman M, Gee JC. Symmetric diffeomorphic image registration with cross-correlation: evaluating automated labeling of elderly and neurodegenerative brain. *Med Image Anal* 2008; **12:** 26-41.

12. Avants BB, Tustison NJ, Song G, Cook PA, Klein A, Gee JC. A reproducible evaluation of ANTs similarity metric performance in brain image registration. *Neuroimage* 2011; **54:** 2033-2044.

13. Nieman BJ, Flenniken AM, Adamson SL, Henkelman RM, Sled JG. Anatomical phenotyping in the brain and skull of a mutant mouse by magnetic resonance imaging and computed tomography. *Physiol Genomics* 2006; **24:** 154-162.

14. Dorr AE, Lerch JP, Spring S, Kabani N, Henkelman RM. High resolution three-dimensional brain atlas using an average magnetic resonance image of 40 adult C57Bl/6J mice. *Neuroimage* 2008; **42:** 60-69.

15. Steadman PE, Ellegood J, Szulc KU, Turnbull DH, Joyner AL, Henkelman RM *et al.* Genetic effects on cerebellar structure across mouse models of autism using a magnetic resonance imaging atlas. *Autism research : official journal of the International Society for Autism Research* 2014; **7:** 124-137.

16. Ullmann JF, Watson C, Janke AL, Kurniawan ND, Reutens DC. A segmentation protocol and MRI atlas of the C57BL/6J mouse neocortex. *Neuroimage* 2013; **78:** 196-203.

17. Zerbi V, Grandjean J, Rudin M, Wenderoth N. Mapping the mouse brain with rs-fMRI: An optimized pipeline for functional network identification. *Neuroimage* 2015; **123:** 11-21.

18. Walker SF. Lateralization of functions in the vertebrate brain: a review. *Br J Psychol* 1980; **71:** 329-367.

19. Duboc V, Dufourcq P, Blader P, Roussigne M. Asymmetry of the Brain: Development and Implications. *Annu Rev Genet* 2015; **49:** 647-672.

20. Ridler TW, Calvard S. Picture thresholding using an iterative selection method. *IEEE Transactions on Systems, Man, and Cybernetics* 1978; **SMC-8:** 630-632.

21. Rocco BR, Lewis DA, Fish KN. Markedly Lower Glutamic Acid Decarboxylase 67 Protein Levels in a Subset of Boutons in Schizophrenia. *Biol Psychiatry* 2015.

22. Ashburner J, Friston KJ. Unified segmentation. *Neuroimage* 2005; **26:** 839-851.

23. Ashburner J. A fast diffeomorphic image registration algorithm. *Neuroimage* 2007; **38:** 95-113.

24. Desikan RS, Segonne F, Fischl B, Quinn BT, Dickerson BC, Blacker D *et al.* An automated labeling system for subdividing the human cerebral cortex on MRI scans into gyral based regions of interest. *Neuroimage* 2006; **31:** 968-980.

25. Frazier JA, Chiu S, Breeze JL, Makris N, Lange N, Kennedy DN *et al.* Structural brain magnetic resonance imaging of limbic and thalamic volumes in pediatric bipolar disorder. *A J Psychiatry* 2005; **162:** 1256-1265.

26. Guilloux JP, Seney M, Edgar N, Sibille E. Integrated behavioral z-scoring increases the sensitivity and reliability of behavioral phenotyping in mice: relevance to emotionality and sex. *J Neurosci Methods* 2011; **197:** 21-31.

27. Wheeler AL, Wessa M, Szeszko PR, Foussias G, Chakravarty MM, Lerch JP *et al.* Further neuroimaging evidence for the deficit subtype of schizophrenia: a cortical connectomics analysis. *JAMA psychiatry* 2015; **72:** 446-455.

28. Csardi G, T N. The igraph software package for complex network research. *InterJournal, Complex Systems* 2006; **1695**.

29. Newman ME, Girvan M. Finding and evaluating community structure in networks. *Phys Rev E Stat Nonlin Soft Matter Phys* 2004; **69:** 026113.

30. Lei D, Li K, Li L, Chen F, Huang X, Lui S *et al.* Disrupted Functional Brain Connectome in Patients with Posttraumatic Stress Disorder. *Radiology* 2015; **276:** 818-827.

31. Shannon P, Markiel A, Ozier O, Baliga NS, Wang JT, Ramage D *et al.* Cytoscape: a software environment for integrated models of biomolecular interaction networks. *Genome Res* 2003; **13:** 2498-2504.

32. Kamada T, Kawai S. An algorithm for drawing general undirected graphs. *Information Processing Letters* 1989; **31:** 7-15.
